# Dielectric-Screened Functionals for Cis–Trans Excitation Energies of Solvated Azobenzene Derivatives: Screening Trends in Comparison with Experiment and Published GW/BSE Calculations

**DOI:** 10.1021/acs.jctc.6c01302

**Published:** 2026-09-21

**Authors:** Aswathy Jayachandran, Barry D. Dunietz

**Affiliations:** 4229 Kent State University, Department of Chemistry and Biochemistry, Kent, Ohio 44242, United States

## Abstract

Accurate modeling of electronically excited states in solution is essential for the rational design of photoactive molecular systems, but requires a reliable treatment of environmental effects. We present a mean-field approach that combines density functional theory with a polarizable continuum model (PCM) to account for environmental dielectric screening, providing an efficient mean-field alternative to higher-cost wavefunction- or many-body-based approaches. The framework employs dielectric-screened range-separated hybrid (SRSH) functionals within PCM to obtain excited states of solvated azobenzene derivatives, a prototypical molecular photoswitch. SRSH–PCM excitation energies better reproduce experiment when the larger static dielectric constant, which also accounts for nuclear polarization, is used to define long-range screening. Using the smaller optical dielectric constant, which represents purely electronic polarization, SRSH–PCM energies align better with previously reported high-quality GW–Bethe–Salpeter equation (GW/BSE) energies [Kshirsagar et al., JCTC 2020]. Our findings demonstrate that mean-field SRSH–PCM provides an effective description of excited-state properties while offering insights into the role of environmental screening and, in particular, the importance of accounting for nuclear contributions to dielectric screening in many-body calculations of solution-phase excitation energies.

## Graphical abstract

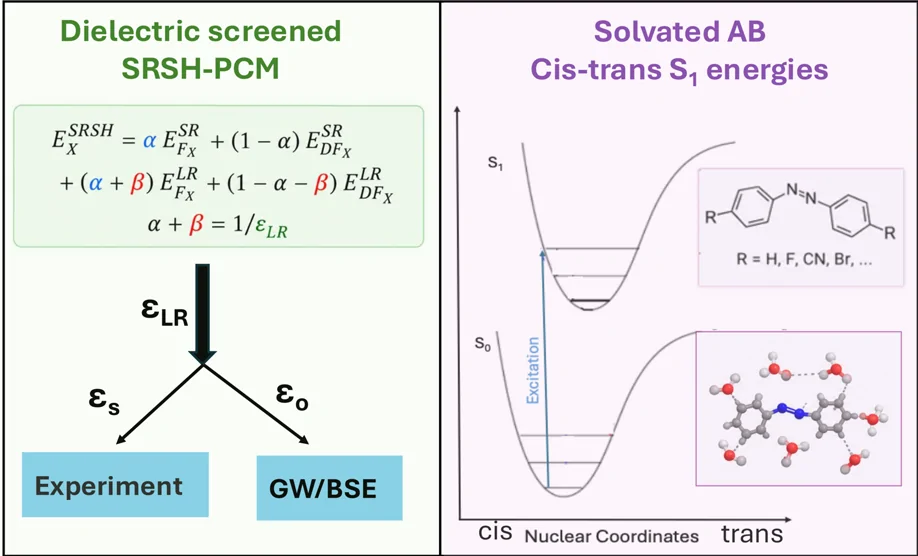

## 1 Introduction

Computational studies can uncover molecular-level insights into the relationships governing optoelectronic properties, guiding the rational design of molecular systems with enhanced functionality and improving our understanding of complex biological processes.^1,2^ Achieving such a predictive insight requires computational frameworks that accurately describe the electronic structure of the active molecular system,^3–8^ while consistently accounting for environmental interactions and conformational flexibility. The goal of predictive electronic-structure descriptions of excited states has motivated the development of high-level many-body methods for modeling photoactive systems, such as the GW–Bethe–Salpeter equation (GW–BSE) approach,^9,10^ which has been successfully applied to the calculation of excitation energies.^11–14^ However, the high computational cost of GW–BSE limits its routine application and restricts its ability to explore conformational flexibility and environmental effects in molecular systems. To address this limitation, density functional theory (DFT), a computationally efficient mean-field framework, can be extended to provide reliable excited-state descriptions in embedded environments. In particular, periodic implementations of range-separated hybrid (RSH) functionals incorporating physically motivated dielectric screening have been applied to study electronic excitations in molecular crystals.^15–18^

As a practical alternative to periodic RSH implementations, dielectric-screened range-separated hybrid (SRSH) functionals combined with a polarizable continuum model (PCM) provide an efficient framework for explicitly accounting for environmental dielectric screening effects.^19–22^ RSH and long-range-corrected (LRC) functionals are well established for overcoming fundamental limitations of DFT in the gas phase.^23–26^ Building on these functionals, the SRSH–PCM framework consistently captures environmental dielectric screening and provides valuable insights into spectral trends in photosystems and organic semiconductors.^20,22,27–41^

To assess the role of dielectric screening within the SRSH–PCM framework, we focus on azobenzene (AB) and its derivatives, which are widely studied for their cis–trans photoisomerization and their potential to function as molecular photoswitches.^42–49^ Molecular photoswitches rely on selective photoactivation to induce reversible conformational changes, enabling applications ranging from drug delivery to molecular electronics.^50^ In this context, the cis–trans excitation energies of AB derivatives provide a useful test set for assessing computational methods designed to describe environment-affected excited states.^8,13^ However, strong spectral overlap between the S_1_ excitations of the cis and trans isomers complicates selective photoactivation,^46^ whereas functionalization provides a route to tune the cis–trans excitation-energy gap and improve switching selectivity.^44,51^

Kshirsagar et al. reported GW–BSE calculations for these AB derivatives, obtaining excitation energies in good agreement with experiment and providing a valuable high-level reference for the present analysis.^13^ Importantly, these published GW–BSE calculations address the effects of environmental screening. Recently, SRSH–PCM has emerged as an effective mean-field approach for obtaining environment-affected excitation energies. In the context of solvated AB systems, it has been shown to accurately reproduce cis–trans excitation energies, in agreement with the average of quantum embedding calculations that explicitly account for molecular-scale environmental effects.^8^ In this study, we examine the SRSH–PCM framework using the AB derivatives test set. We assess its performance in reproducing experimental absorption energies and analyze the impact of the dielectric screening setup, including comparison to the published GW–BSE excitation energies of Kshirsagar et al.^13^ Analysis of the screening trends relative to experimental observations and the published GW–BSE results provides insight into the role of environmental dielectric screening in calculations of solution-phase excitation energies.

## 2 Computational Details

RSH functionals are formulated within a generalized Kohn–Sham (GKS) framework for the exchange–correlation energy (*E*_XC_).^16,19–21,52^ In this approach, the electron–electron Coulomb interaction is partitioned using the error function:

1 $$\frac{1}{r}=\frac{\alpha +\beta \mathrm{erf}\left(\omega r\right)}{r}+\frac{1-\left(\alpha +\beta \mathrm{erf}\left(\omega r\right)\right)}{r}$$

where *r* is the electron–electron separation, and α, β, and ω are functional parameters. The range-separated GKS exchange–correlation functional is obtained by assigning the first and second terms in eq 1 to exact exchange (*F*_X_) and local density exchange (*DF*_X_), respectively. In the limits *r* → 0 and *r* → ∞, this partition yields fractions of exact exchange equal to α and α + β for the short-range (SR) and long-range (LR) interactions, respectively.

More specifically, the GKS functional takes the form

2 $$\begin{array}{l} E_{XC}^{SRSH}=\alpha E_{F_{X}}^{SR}+\left(1-\alpha \right)E_{DF_{X}}^{SR}+\left(\alpha +\beta \right)E_{F_{X}}^{LR}\\ +\left(1-\alpha -\beta \right)E_{DF_{X}}^{LR}+E_{DF_{C}}, \end{array}$$

where the SR and LR contributions are indicated by the superscripts, and the correlation contribution is treated entirely at the density-functional level (*DF*_*C*_). The range-separation parameter ω controls the switching between SR and LR interactions, as described next. In the gas phase, ω is determined by optimal tuning (OT), which enforces Koopmans’ conditions relating the HOMO energy to the ionization potential (IP) for both the neutral and anionic states.^53,54^

As commonly implemented, the anionic-state condition is replaced by relating the neutral-state LUMO energy to its electron affinity (EA).^54–60^ Accordingly, the optimal ω is obtained by minimizing

3 $$J\left(\omega \right)={\left[\varepsilon_{HOMO}\left(\omega \right)+IP\left(\omega \right)\right]}^{2}+{\left[\varepsilon_{LUMO}\left(\omega \right)+EA\left(\omega \right)\right]}^{2}$$

where ε_HOMO_ and ε_LUMO_ denote the highest occupied and lowest unoccupied molecular orbital energies, respectively, and the IP and EA are obtained from total energy differences of the neutral, cationic, and anionic species.

To describe condensed-phase effects, a PCM is typically employed. The PCM modifies the ionization energies according to the dielectric screening of the environment, with the oxidation potential (OP) and reduction potential (RP) considered in place of the IP and EA. In standard DFT calculations without explicit environmental screening, however, PCM-calculated frontier orbital energies remain close to their gas-phase values. The SRSH framework overcomes this limitation by explicitly including dielectric screening in the functional expression, yielding frontier orbital energies that align with the corresponding condensed-phase ionization energies.^20^ In SRSH functionals, dielectric effects are incorporated by modifying the LR limit in eq 2 as determined by the environmental dielectric constant ε, where

4 $$\alpha +\beta =\frac{1}{\varepsilon }$$

Most SRSH implementations set β according to eq 4, ensuring that the asymptotic fraction of exact exchange is screened by the surrounding medium.^8,22,29,34,37,39,61^ Parameter retuning can then be performed using SRSH–PCM energies either by scanning α, while keeping ω fixed, or—as implemented here—by tuning ω through minimization of the error measure in eq 3. For larger conjugated systems, such PCM-based retuning is generally less critical. Excitation energies were calculated using full linear-response time-dependent density functional theory (TDDFT), without invoking the Tamm–Dancoff approximation (TDA). All occupied–virtual transitions were included in the response calculation.

A state-specific (SS)^62–64^ non-equilibrium PCM correction is evaluated using the perturbative formulation implemented in Q-Chem.^65–67^ The excited-state density, constructed within linear-response theory, is used to relax the PCM reaction field, yielding the relaxed ptSS correction.^65,66,68–70^ This relaxed ptSS form remains an approximation of the full SS treatment, but is less computationally demanding. In all PCM calculations, the ground-state is equilibrated with the full solvent polarization, parameterized by ε_*s*_, while for vertical excitations only the fast electronic component, parameterized by ε_*o*_, responds to the change in solute electronic density, with the slow nuclear component remaining frozen at its ground-state value. This partitioning yields the non-equilibrium SS-PCM correction for vertical excitations.^65,66,68–70^ The corresponding perturbative linear-response (ptLR) contribution was also evaluated. Because ptSS and ptLR describe complementary contributions to the non-equilibrium solvent response and are evaluated relative to the same zeroth-order TDDFT/PCM reference, they can be combined without double counting; the resulting correction is denoted pt­(SS+LR).

Below, we report SRSH–PCM calculations performed with varied α values, where the long-range exchange weight of the functional is set by ε_LR_, ranging from the smaller optical dielectric constant ε_*o*_ to the larger static dielectric constant ε_*s*_. Here, the use of the dielectric constant to define the functional screening is distinct from its role in the non-equilibrium PCM treatment of the excitation. The AB molecules considered in this study are shown in Figure 1, with the experimental reference values corresponding to the absorption-band maxima assigned to the *n*π* transitions. The calculated excitation energies are also compared with previously published GW–BSE calculations by Kshirsagar et al.^13^ These GW–BSE excitation energies were obtained starting from a semi-local functional with PCM response included to account for solvent effects. The exact-exchange fraction α of the global hybrid PBEh functional was then tuned such that the Kohn–Sham HOMO energy matched the corresponding GW quasiparticle HOMO energy, mitigating the starting-point dependence of the GW–BSE calculation. Excitation energies were then calculated using this tuned PBEh starting point. This procedure achieved mean absolute deviations as low as 0.06 eV relative to the experimental values reported in that study.

**Figure 1:**
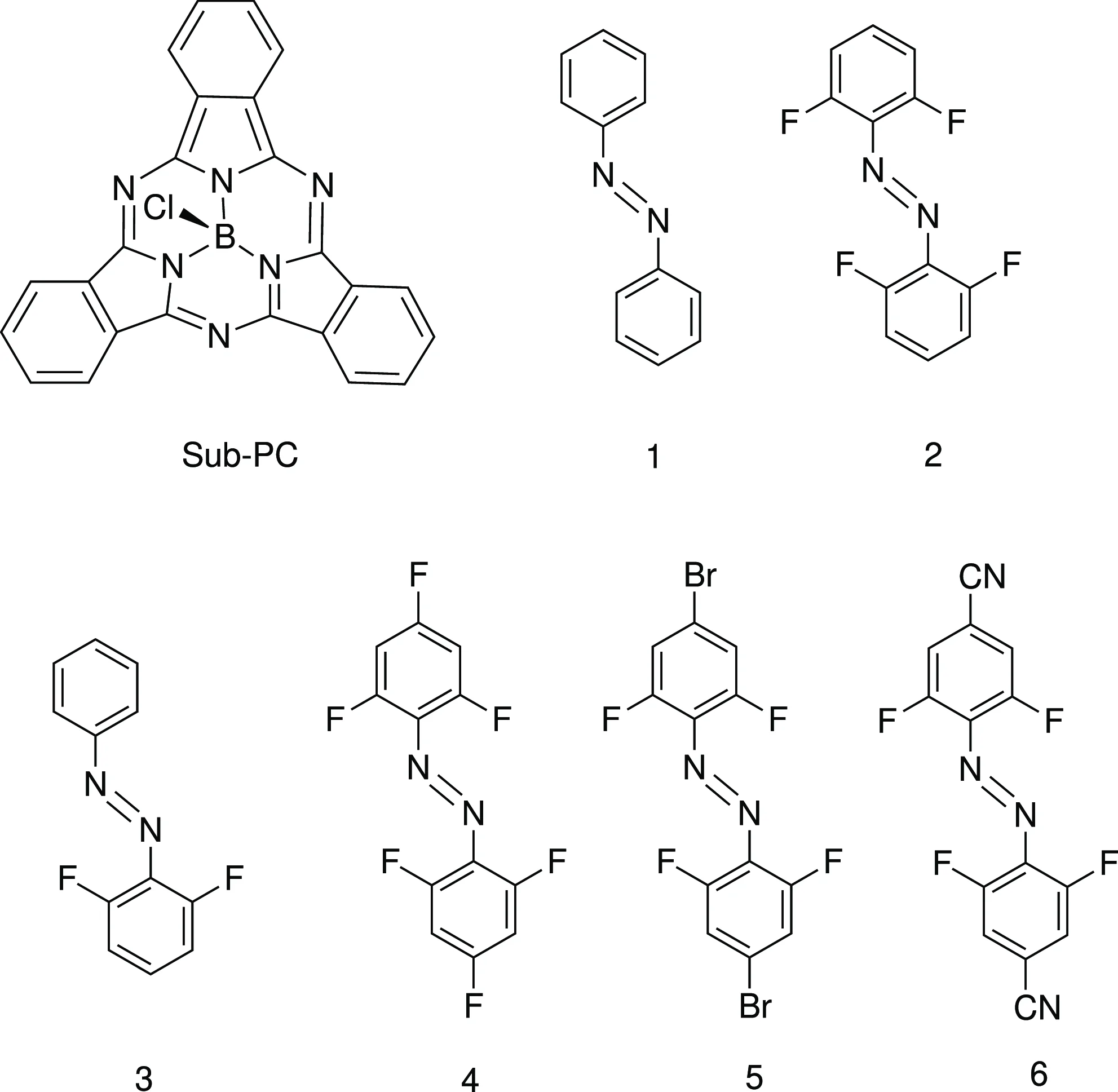
Structures of SubPC and the AB derivatives considered in this study. SubPC is included as a representative system to illustrate general trends in orbital gaps. The set consists of cis and trans isomers of functionalized AB derivatives, with excitation energies in solution available from experiment and published GW–BSE calculations. SubPC is included to illustrate general trends in orbital gaps.

All PCM calculations—including geometry optimizations and excited-state calculations—were performed using the conductor-like PCM^71^ with acetonitrile as the solvent (dielectric constants ε_*s*_ = 37.5 and ε_*o*_ = 1.796) and the cc-pVTZ basis set, unless noted otherwise. Geometry optimizations employed the dispersion-corrected ωB97X-D functional.^72^ SRSH–PCM results obtained with the 6-31G* basis set are also reported to demonstrate basis-set convergence. All calculations were performed using Q-Chem 6.0.2.

## 3 Results

### 3.1 SubPC Preliminary Benchmark

#### 3.1.1 Frontier Orbital Energies and Gaps

Before presenting the main analysis of excited-state energies for AB derivatives, we first illustrate the general trends in ground-state orbital gaps using the subphthalocyanine (SubPC) molecule (Figure 1). SubPC, being a larger conjugated system than AB, exhibits a tuned range-separation (RS) parameter that is less sensitive to variations in the dielectric environment, providing a useful system for demonstrating these energy-gap trends. For this analysis, we consider the crystalline form of SubPC, with dielectric constants ε_*s*_ = 24.2 and ε_*o*_ = 3.1, and explore the impact of LR dielectric screening, ε_LR_, on its orbital gaps.

The gas-phase IP and EA, condensed-phase OP and RP, and frontier molecular orbital (MO) energies of SubPC calculated using the 6-31G* basis set are listed in Table 1. The upper part of the table reports gas-phase RSH results. SRSH–PCM results are reported for LR dielectric screening corresponding to the optical dielectric constant (ε_*o*_), static dielectric constant (ε_*s*_), and their average (ε_ave_), together with results obtained by retuning ω for ε_LR_ = ε_*s*_ and ε_ave_. The RSH and corresponding SRSH functionals are primarily evaluated for α = 0.20 and 0.35, which are widely used in previous studies. For completeness, we also report the RSH–PCM limit (ε_LR_ = 1) obtained using the gas-phase tuned ω without dielectric-dependent retuning, together with results for α = 0.00 and 0.45. Notably, although the tuned RS parameter decreases with increasing α, the RSH IP and EA remain essentially unchanged at approximately 6.6 and 1.4 eV, respectively, across the considered α values. These RSH-calculated IP and EA values are in good agreement with the corresponding HOMO and LUMO energies, reflecting the success of the gas-phase tuning. A smaller RS parameter associated with a larger α value indicates a longer switching distance, compensating for the increased exact-exchange contribution.

**Table 1:** Ground-State Properties of SubPC in the Gas Phase and in the Condensed Phase (ε_*s*_ = 24.2, ε_*o*_ = 3.1) Using the 6-31G* Basis Set^a^

method	α	ε_LR_	ω	IP	EA	*H*	*L*	*J*
Gas ^b^	0.00	1	178	6.63	1.36	–6.64	–1.37	1.0 × 10^–7^
RSH	0.20	1	142	6.62	1.40	–6.63	–1.41	3.0 × 10^–7^
	0.35	1	110	6.59	1.44	–6.61	–1.46	6.0 × 10^–7^
	0.45	1	84	6.56	1.48	–6.58	–1.50	1.1 × 10^–6^
Condensed	0.00	1	178	5.33	2.87	–6.71	–1.48	5.2 × 10^–3^
RSH–PCM	0.20	1	142	5.33	2.93	–6.71	–1.54	5.2 × 10^–3^
	0.35	1	110	5.31	3.00	–6.70	–1.59	5.3 × 10^–3^
	0.45	1	84	5.28	3.05	–6.68	–1.64	5.3 × 10^–3^
SRSH–PCM	0.00	ε_s_	178	5.29	2.81	–5.06	–3.09	1.8 × 10^–4^
		ε_ave_	178	5.30	2.81	–5.11	–3.03	1.1 × 10^–4^
		ε_o_	178	5.31	2.82	–5.54	–2.62	1.2 × 10^–4^
	0.20	ε_s_	142	5.33	2.88	–5.24	–3.00	3.1 × 10^–5^
		ε_ave_	142	5.33	2.88	–5.29	–2.95	9.3 × 10^–6^
		ε_o_	142	5.33	2.89	–5.67	–2.57	2.9 × 10^–4^
	0.35	ε_s_	110	5.33	2.95	–5.44	–2.86	2.6 × 10^–5^
		ε_ave_	110	5.33	2.95	–5.48	–2.82	5.4 × 10^–5^
		ε_o_	110	5.32	2.96	–5.81	–2.49	6.2 × 10^–4^
	0.45	ε_s_	84	5.30	3.01	–5.62	–2.70	2.8 × 10^–4^
		ε_ave_	84	5.30	3.02	–5.66	–2.66	3.4 × 10^–4^
		ε_o_	84	5.29	3.03	–5.94	–2.39	1.1 × 10^–3^
(retuned)	0.2	ε_s_	63	5.33	2.89	–5.34	–2.90	4.0 × 10^–7^
		ε_ave_	80	5.34	2.88	–5.35	–2.89	3.0 × 10^–7^
	0.35	ε_s_	160	5.33	2.94	–5.35	–2.95	4.0 × 10^–7^
		ε_ave_	221	5.33	2.93	–5.34	–2.95	9.0 × 10^–7^
	0.45	ε_s_	211	5.33	2.96	–5.34	–2.97	4.0 × 10^–7^
		ε_ave_	282	5.32	2.94	–5.33	–2.96	7.0 × 10^–7^
(Exp.^73^)				5.50	3.40			

a Listed are the ionization potential (IP) and electron affinity (EA) in the gas phase, or the oxidation (OP) and reduction (RP) potentials in the condensed phase, HOMO and LUMO energies (in eV), as well as the tuned range-separation (RS) parameter ω and the error measure *J*, evaluated for various RSH setups defined by different values of α and ε_LR_.

b Experimental gas phase values are not available.

In the condensed phase, the RSH–PCM-calculated OP and RP energies vary by only 0.05 and 0.07 eV, respectively, across the α values considered here and remain in reasonable agreement with the available experimental values. More generally, the OP and RP energies are only weakly affected by α, RS retuning in PCM, or ε_LR_, whereas the frontier orbital energies show a much stronger dependence on these aspects of the functional setup. The frontier orbital energies undergo substantial renormalization upon introduction of dielectric screening in SRSH–PCM. Even without retuning the range-separation parameter ω, the resulting HOMO and LUMO energies become reasonably consistent with the corresponding electron removal and addition energies (OP/RP). This agreement provides a ground-state measure of the effect of dielectric screening on the frontier orbital energies. The extent of this effect increases with increasing ε_LR_. In contrast, for ε_LR_ = 1, corresponding to the RSH–PCM level, the orbital energies remain close to their gas-phase values, in agreement with earlier observations,^20^ and retuning in this case appears to fail.

The PCM-retuned RS parameter, ω, exhibits a complex, non-monotonic dependence on both α and ε_LR_. For the lower value of α (i.e., α = 0.2), the tuned ω decreases relative to the gas-phase value, becoming very small upon tuning for α = 0.0. In contrast, for higher values of α (0.35 and 0.45), the tuned ω increases. In all cases, the tuned ω increases as the dielectric screening, ε_LR_, is reduced from ε_*s*_ to ε_ave_.

### 3.2 Azobenzene (AB) Derivatives

#### 3.2.1 Frontier Orbital Energies and Gaps

We first examine the effect of long-range dielectric screening on the frontier orbital energies of the AB derivatives. The *cis*-1-AB isomer is considered in detail, with the orbital energies and corresponding IP/EA values summarized in Table 2. Unless noted otherwise, acetonitrile is used as the solvent. The calculations employ RSH–PCM and SRSH–PCM functionals with α = 0.20, 0.35, and 0.45, with ε_LR_ set to either ε_*o*_ or ε_*s*_; RSH–PCM corresponds to ε_LR_ = 1. The corresponding *trans*-isomer results are provided in Table S1.

**Table 2:** Ground-State Properties of *cis*-1-AB in the Gas Phase and in Acetonitrile (ε_s_ = 37.5, ε_o_ = 1.796) Using the cc-pVTZ Basis Set^a^

method	α	ε_LR_	ω	IP	EA	H	L	*J*
gas	0.20	1	184	7.94	0.24	–7.90	–0.24	2.4 × 10^–6^
RSH	0.35	1	140	8.02	0.24	–7.98	–0.22	2.7 × 10^–6^
	0.45	1	103	8.08	0.24	–8.02	–0.22	7.5 × 10^–6^
condensed	0.20	1	184	6.29	2.32	–8.10	–0.40	9.4 × 10^–3^
RSH–PCM	0.35	1	140	6.37	2.35	–8.16	–0.39	9.7 × 10^–3^
	0.45	1	103	6.43	2.38	–8.19	–0.40	9.7 × 10^–3^
SRSH–PCM	0.20	ε_s_	184	6.03	2.33	–5.79	–2.56	1.5 × 10^–4^
		ε_o_	184	6.17	2.32	–7.04	–1.39	2.2 × 10^–3^
	0.35	ε_s_	140	6.21	2.34	–6.29	–2.17	4.8 × 10^–5^
		ε_o_	140	6.30	2.35	–7.31	–1.21	3.2 × 10^–3^
	0.45	ε_s_	103	6.36	2.37	–6.75	–1.80	7.1 × 10^–4^
		ε_o_	103	6.40	2.37	–7.53	–1.03	4.3 × 10^–3^
(retuned)	0.20	ε_s_	50	6.07	2.33	–6.07	–2.31	1.0 × 10^–7^
	0.35	ε_s_	192	6.17	2.34	–6.13	–2.31	2.5 × 10^–6^
	0.45	ε_s_	254	6.20	2.36	–6.16	–2.31	5.1 × 10^–6^

a Listed are the ionization potential (IP) and electron affinity (EA) in the gas phase, or the oxidation (OP) and reduction (RP) potentials in solution, HOMO and LUMO energies (in eV), as well as the tuned range-separation (RS) parameter ω and the error measure *J*, evaluated for the RSH and SRSH–PCM setups defined by the indicated values of α and ε_LR_. Corresponding trans-1-AB results are listed in Table S1, and, for completeness, 6-31G* results are provided in Tables S2 and S3.

Even before retuning, the effect of dielectric screening on the SRSH–PCM orbital energies increases with the strength of the long-range screening, such that the orbital energies obtained with ε_*s*_ are generally closer to the corresponding OP and RP values than those obtained with the smaller ε_*o*_. This systematic dependence provides ground-state evidence for the role of dielectric screening in determining the frontier orbital energies. The retuning-induced orbital-energy shifts are also larger than in the SubPC case, consistent with the smaller molecular size and reduced conjugation of the AB system.

The OP and RP values themselves are only weakly affected by retuning. Compared to RSH–PCM, the retuned SRSH–PCM IP changes by about 0.2 eV, while the change in the EA remains below 0.1 eV. Across the considered range of α values, the IP/HOMO energies vary within approximately 0.1 eV, with an even narrower variation for the EA/LUMO energies; similar trends are found for the trans isomer (Table S1). The smaller-basis-set results, shown in Tables S2 and S3, exhibit the same trends, with somewhat larger differences for the EA/LUMO values.

Figure 2 illustrates the frontier orbital energy trends for ε_LR_ = ε_*s*_. Without PCM retuning, the frontier orbital energies and HOMO–LUMO gap depend strongly on α. PCM retuning largely removes this dependence, yielding frontier orbital energies that vary by only ∼0.1 eV and a HOMO–LUMO gap that changes by less than 0.1 eV across the range of α values considered. In particular, for *cis*-1-AB, the SRSH–PCM orbital energies at α = 0.35 already agree with the corresponding OP and RP values to within about 0.1 eV before PCM retuning. This value of α is therefore used for all subsequent SRSH–PCM calculations unless otherwise noted.

**Figure 2:**
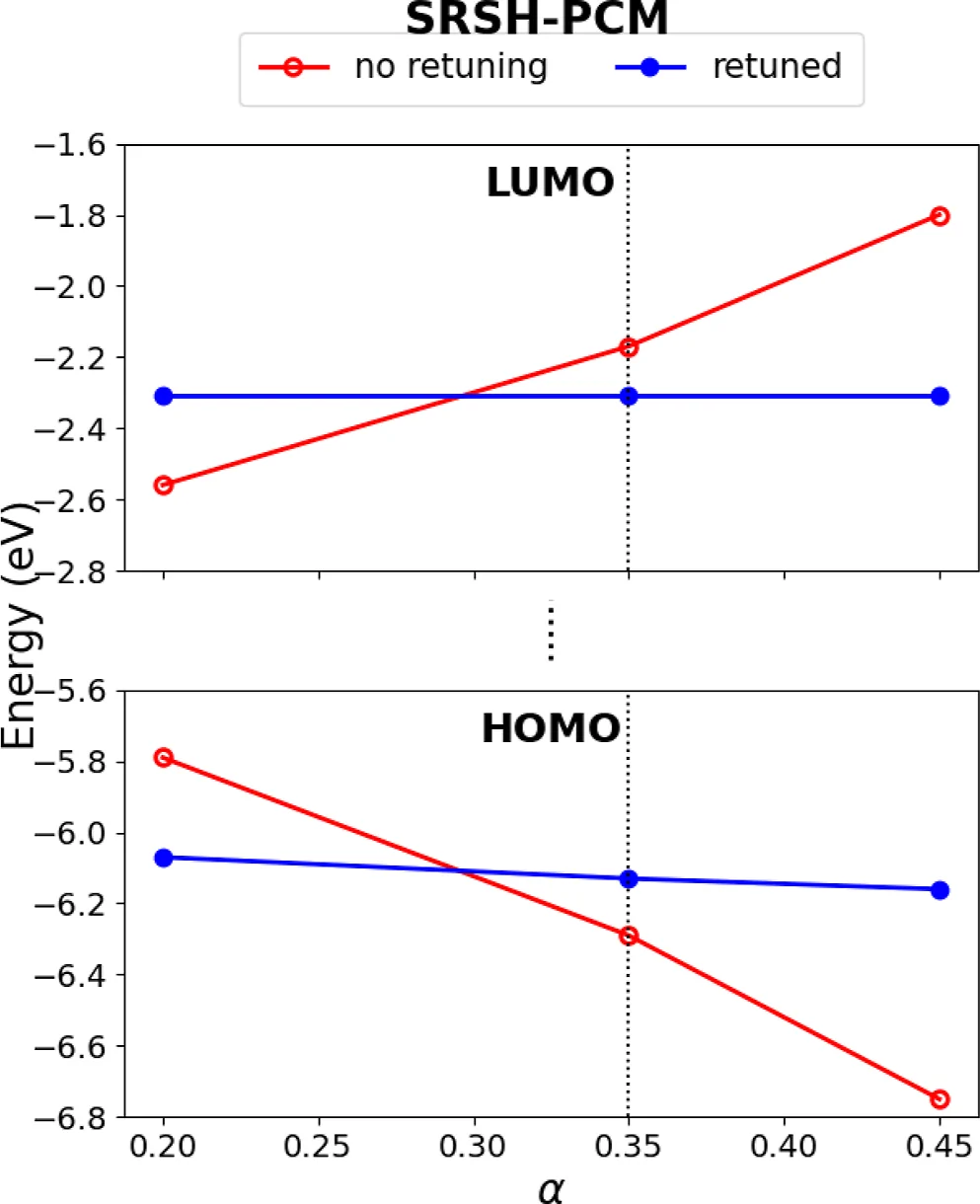
HOMO and LUMO energies of *cis*-1-AB obtained at the SRSH–PCM level with ε_LR_ = ε_s_ for varied α values, with and without PCM retuning of ω. The ionization potential (IP) in PCM is 6.37 eV for RSH and 6.21 eV for SRSH. For reference the gas-phase IP and EA are 8.02 and 0.24 eV, respectively.

#### 3.2.2 S_1_ Excitation Energies

We next examine the effect of long-range dielectric screening on the *S*_1_ excitation energies of the functionalized AB derivatives. This effect is first analyzed in detail for the *cis*- and *trans*-1-AB isomers. As shown in Figure 3, the calculated *S*_1_ energies decrease systematically with increasing screening, spanning the range between the lower-screening (ε_LR_ = ε_*o*_) and higher-screening (ε_LR_ = ε_*s*_) limits. The experimental reference values correspond to the maxima of the absorption bands assigned to the *n*π* transitions. Our analysis therefore focuses on the systematic screening trends across the full set of the AB derivatives rather than on the significance of small deviations from the reference values. At the higher-screening limit (ε_LR_ = ε_*s*_), the calculated *S*_1_ energies are lower than but close to the experimental values for both isomers. At the lower-screening limit (ε_LR_ = ε_*o*_), the *S*_1_ energies increase and approach the published BSE values of Kshirsagar et al.

**Figure 3:**
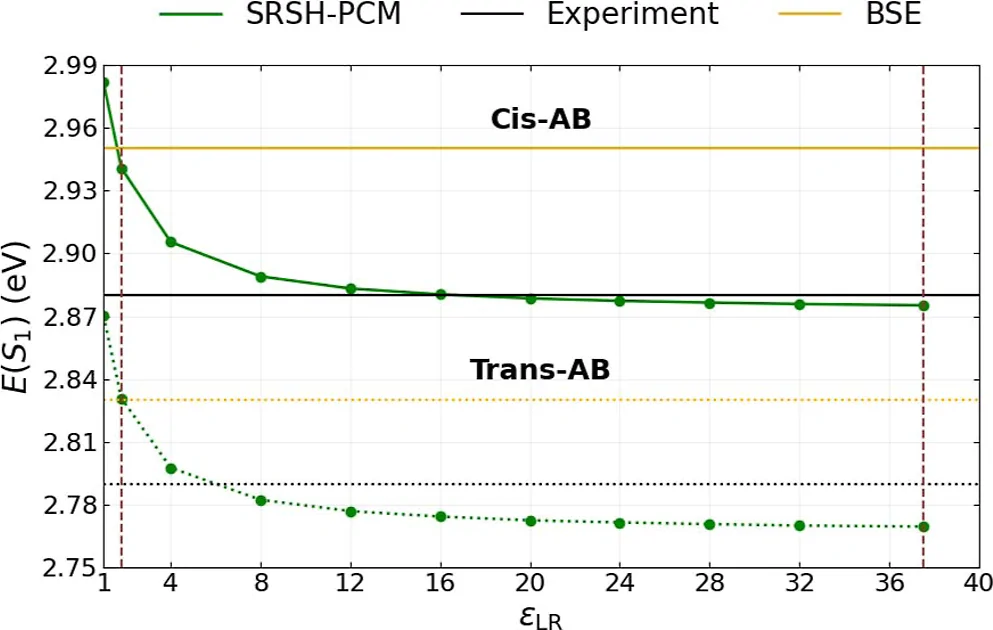
*S*_1_ excitation energies of *cis*-AB (solid line) and *trans*-AB (dotted lines) at ε_LR_ values ranging from ε_o_ to ε_s_, compared with experimental measurements and published BSE values.^13^ The energies show better agreement with experiment at the larger dielectric constant (ε_s_) and with BSE at the smaller dielectric constant (ε_o_). Vertical dotted lines indicate ε_o_ = 1.796 and ε_s_ = 37.5.

Having established the dielectric-screening trends for the 1-AB isomers, we now turn to the complete set of fluorinated AB derivatives. Table 3 reports the HOMO, LUMO, OP, and RP energies obtained at the SRSH–PCM level using α = 0.35, ε_LR_ = ε_s_, and the corresponding PCM-retuned range-separation parameter. The resulting frontier orbital energies are well aligned with the OP and RP values across the series, extending the ground-state screening behavior established above for *cis*-1-AB. The improved alignment of the frontier orbital energies with the OP and RP values obtained with the larger long-range screening provides an independent ground-state indication of the role of dielectric screening. To enable comparison of the two screening limits in the excited-state analysis below, Table 4 presents the corresponding orbital energies obtained with ε_LR_ = ε_*o*_, for which the gas-phase tuned ω is employed. Gas-phase RSH results used to establish these ω values are provided in Table S5.

**Table 3:** Frontier Orbital energies—HOMO (H) and LUMO (L), OPs and RPs (eV)—for the AB Derivatives Solvated in Acetonitrile, Calculated at the SRSH–PCM Level with ε_LR_ = ε_*s*_, α = 0.35, and the PCM-Tuned RS Parameter and Error Measure *J*, Using the cc-pVTZ Basis Set^a^

AB	ω	OP	RP	*H*	*L*	*J*
1 cis	192	6.17	2.34	–6.13	–2.31	2.5 × 10^–6^
trans	193	6.42	2.73	–6.40	–2.70	8.0 × 10^–7^
2 cis	171	6.74	2.74	–6.72	–2.70	1.8 × 10^–6^
trans	175	6.56	2.90	–6.56	–2.88	1.0 × 10^–7^
3 cis	180	6.47	2.53	–6.44	–2.49	2.5 × 10^–6^
trans	184	6.53	2.82	–6.52	–2.80	4.0 × 10^–7^
4 cis	166	6.74	2.77	–6.71	–2.73	2.8 × 10^–6^
trans	168	6.57	2.89	–6.55	–2.86	5.0 × 10^–7^
5 cis	169	6.75	2.92	–6.72	–2.87	2.3 × 10^–6^
trans	173	6.59	3.11	–6.58	–3.08	4.0 × 10^–7^
6 cis	165	7.17	3.28	–7.16	–3.26	3.0 × 10^–7^
trans	171	6.98	3.55	–6.99	–3.54	2.0 × 10^–7^

a Values obtained with the 6-31G* basis set are provided in Table S4. Gas-phase RSH energies are provided in Table S5.

**Table 4:** Frontier Orbital Energies (eV)—HOMO (H) and LUMO (L)—for the AB Derivatives Solvated in Acetonitrile, Calculated at the SRSH–PCM Level Using ε_LR_ = ε_*o*_, α = 0.35, and the Gas-Phase Tuned ω, with the cc-pVTZ Basis Set^a^

AB	ω	*H*	*L*
1 cis	140	–7.31	–1.21
trans	136	–7.55	–1.68
2 cis	141	–7.84	–1.64
trans	133	–7.67	–1.90
3 cis	140	–7.58	–1.40
trans	134	–7.65	–1.79
4 cis	142	–7.81	–1.67
trans	135	–7.64	–1.89
5 cis	128	–7.78	–1.86
trans	122	–7.63	–2.14
6 cis	131	–8.23	–2.27
trans	123	–8.06	–2.61

a Gas-phase RSH orbital energies, IPs, and EAs are listed in Table S5, and 6-31G* energies are provided in Table S6.

The *S*_1_ excitation energies for the full set are listed in Table 5 and compared with experimental values and the published BSE results of Kshirsagar et al.^13^ The higher-screening results (ε_LR_ = ε_*s*_) are systematically lower in energy and generally show closer agreement with experiment than the lower-screening results. In contrast, the lower-screening results (ε_LR_ = ε_*o*_), which more closely correspond to the electronic-screening character underlying the BSE treatment, yield systematically higher excitation energies and show closer agreement with the published BSE values. The corresponding 6-31G* excitation energies are systematically higher than the cc-pVTZ values by only 0.00–0.03 eV across the series (Table S7), confirming that the excitation energies are well converged with respect to the basis set. The excitation energies and their corresponding deviations from experiment and the BSE values are shown in Figure 4 and analyzed further below.

**Table 5:** *S*_1_ Excitation Energies (eV) for the AB Derivatives Calculated at the SRSH–PCM Level Using ε_LR_ Set to either ε_*s*_ or ε_*o*_, α = 0.35, and the cc-pVTZ Basis Set^a^

AB isomer	ε_LR_ =		Exp^74^	BSE^13^
	ε_s_	ε_o_		
1 cis	2.88	2.93	2.88	2.95
trans	2.77	2.82	2.79	2.83
2 cis	2.97	3.01	2.99	3.03
trans	2.68	2.72	2.72	2.67
3 cis	2.94	2.99	2.96	3.01
trans	2.74	2.78	2.75	2.72
4 cis	2.97	3.01	2.99	3.02
trans	2.73	2.77	2.76	2.81
5 cis	2.94	2.99	2.94	2.95
trans	2.64	2.69	2.71	2.75
6 cis	2.92	2.98	2.95	3.06
trans	2.54	2.60	2.64	2.53

a Corresponding 6-31G* energies are provided in Table S7, showing weak basis-set dependence of the calculated excitation energies.

**Figure 4:**
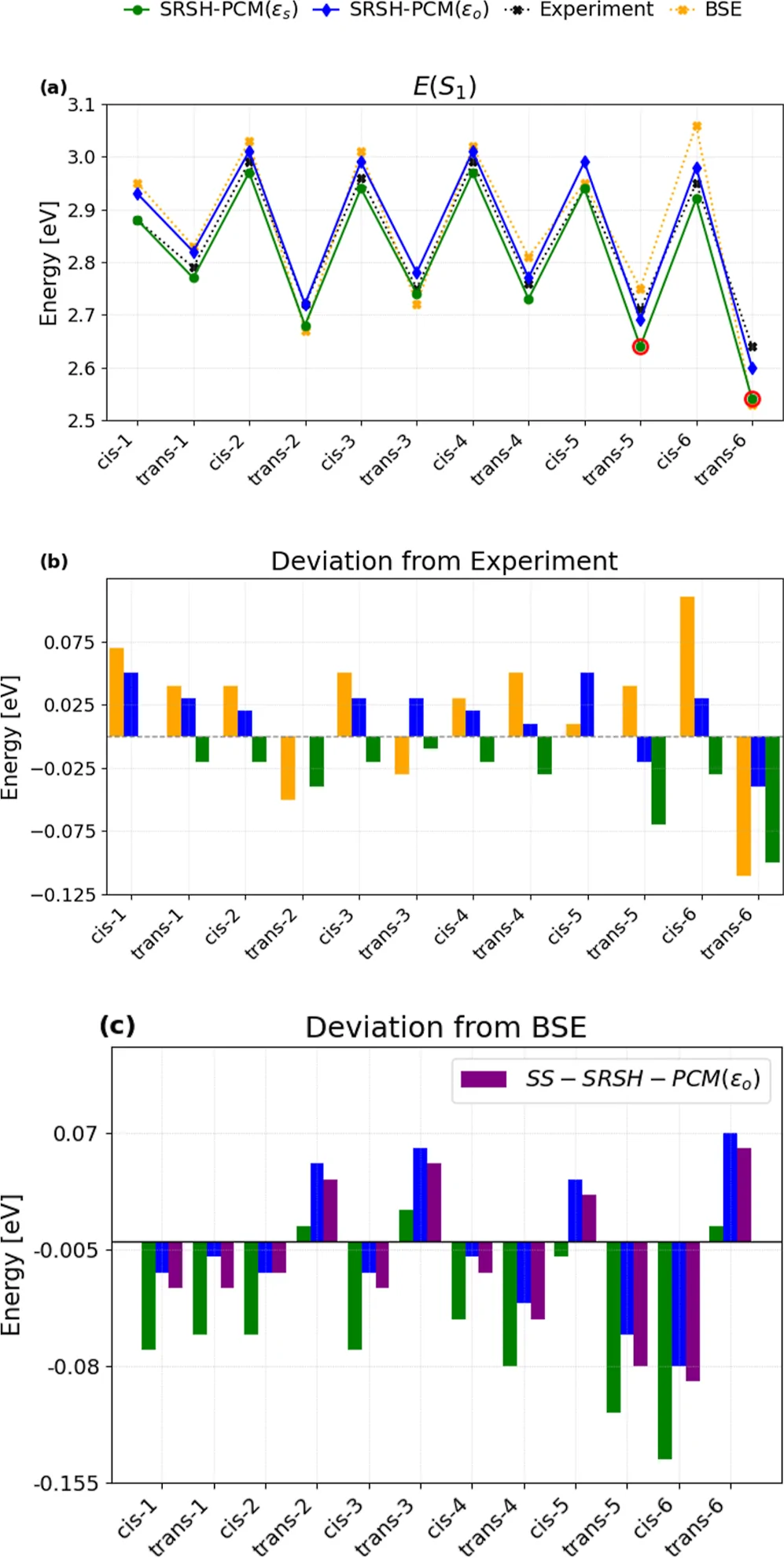
(a) SRSH–PCM calculated *S*_1_ excitation energies of AB derivatives using ε_LR_ = ε_s_ and ε_LR_ = ε_o_, compared to experimental measurements and published BSE values. The larger dielectric constant (ε_s_) generally yields energies closer to experiment, whereas the smaller dielectric constant (ε_o_) generally yields energies closer to the published BSE values. (b) Differences of SRSH–PCM *S*_1_ energies from experimental values for both choices of ε_LR_, showing that using ε_*s*_ generally yields energies closer to experiment than ε_*o*_. (c) Deviations of SRSH–PCM energies from BSE results, including the ptSS-corrected energies for ε_LR_ = ε_*o*_, which systematically reduce the discrepancy with BSE.

For completeness, we report additional combinations of range-separation tuning as a control check, in which the range-separation parameters are crossed with respect to the long-range dielectric screening. Specifically, SRSH–PCM excitation energies obtained with ε_LR_ = ε_s_ using the gas-phase tuned ω, and with ε_LR_ = ε_o_ using the PCM-tuned ω, are provided in Tables S8 and S9, respectively. The resulting excitation energies remain within 0.02 eV of those obtained with the original tuning protocols, confirming that the observed excitation-energy trends are primarily due to the choice of ε_LR_ rather than the tuning procedure.

Further examining the trends in Figure 4, we find that the SRSH–PCM *S*_1_ excitation energies obtained with ε_LR_ = ε_s_ are generally close to the experimental values, although the calculated energies tend to underestimate them. For the cis isomers, the deviations are at most −0.02 eV, with the exception of *cis*-6-AB, which deviates by −0.03 eV. The trans isomers exhibit larger underestimations than their corresponding cis isomers, with the 3-AB cis/trans pair being the only slight exception to this trend. The largest underestimations occur for *trans*-5-AB and *trans*-6-AB, whose excitation energies are lower than experiment by 0.07 and 0.10 eV, respectively, compared with at most 0.04 eV for the remaining derivatives. Only for these two outliers do the lower-screening results (ε_LR_ = ε_o_) provide better agreement with experiment. With the exception of *trans*-5-AB, the ε_LR_ = ε_s_ SRSH–PCM energies are closer to the experimental values than the corresponding published BSE energies. The origin of the larger deviations for *trans*-5-AB and *trans*-6-AB is examined next.

**Table 6:** Calculated Polarizability (P) and Dipole Moment (μ) of the Considered AB Derivatives at the SRSH–PCM Level Using the cc-pVTZ Basis Set

AB	*P*	μ
1 cis	207.864	4.347
trans	239.116	0.132
2-cis	202.377	7.270
trans	232.861	0.258
3-cis	204.504	5.787
trans	234.775	0.949
4-cis	202.160	4.392
trans	234.018	0.187
5-cis	265.912	4.094
trans	312.918	0.170
6-cis	254.873	2.058
trans	295.264	0.044

To understand the larger deviations observed for several of the trans isomers, we consider the polarizabilities of the AB derivatives. The trans isomers exhibit larger polarizabilities than their cis counterparts and, correspondingly, tend to show larger underestimations of the experimental excitation energies. These trends are most pronounced for the *trans*-5-AB and *trans*-6-AB isomers, which exhibit the highest polarizabilities in the series (313 and 295 a.u., respectively, compared with 239 a.u. for *trans*-1-AB; see Table 6 for the complete list). For these highly polarizable systems, the results suggest that the larger static dielectric constant may overestimate the effective long-range screening relevant for the excitation, leading to lower *S*_1_ excitation energies with ε_LR_ = ε_*s*_. Lowering ε_LR_ to ε_*o*_ reduces the underestimation to −0.02 and −0.04 eV for *trans*-5-AB and *trans*-6-AB, respectively. More generally, the experimental excitation energies of the remaining trans isomers fall between the values obtained with ε_LR_ = ε_*s*_ and ε_LR_ = ε_*o*_, consistent with the observed dependence on long-range screening and their generally higher polarizabilities relative to the corresponding cis isomers.

We next consider the improved agreement with the published BSE values obtained using the lower optical dielectric screening, ε_LR_ = ε_*o*_. For the cis isomers, the SRSH–PCM excitation energies agree with the BSE values to within 0.02 eV, with only two exceptions: *cis*-5-AB, which overestimates the BSE value by 0.04 eV, and *cis*-6-AB, which underestimates it by 0.08 eV. The BSE excitation energies lie above experiment for all cis isomers except *cis*-5-AB, with *cis*-6-AB showing by far the largest positive deviation (0.11 eV). Notably, the largest difference between the BSE and SRSH–PCM energies occurs for the isomer for which the published BSE value also deviates most strongly from experiment. Overall, the systematic agreement between SRSH–PCM with ε_LR_ = ε_*o*_ and the published BSE values is consistent with the use of the optical dielectric response to describe the fast electronic component of solvent screening in the GW/BSE non-equilibrium embedding treatment. For the trans isomers, the SRSH–PCM excitation energies agree with the published BSE values within 0.01–0.07 eV, with the largest deviation again occurring for *trans*-6-AB. *Trans*-2-AB, *trans*-3-AB, and *trans*-6-AB are the only systems for which the BSE values lie below experiment, contrary to the overall trend across the series. Consistent with this behavior, only for these three systems are the higher-screening results obtained with ε_LR_ = ε_*s*_ closer to the BSE values than the corresponding ε_LR_ = ε_*o*_ results.

We next assess the perturbative linear-response (ptLR) and state-specific (ptSS) corrections,^65,68,69,75^ listed in Table 7. Both contributions are small. For ε_LR_ = ε_*s*_, the ptLR corrections range from approximately −15 to −21 meV. The ptSS corrections are smaller for the cis isomers, ranging from −7 to −8 meV, whereas those for the trans isomers are larger in magnitude, ranging from −12 to −18 meV. The variations in the ptLR and ptSS contributions partially offset each other, resulting in a relatively narrow range of approximately −27 to −33 meV for the combined pt­(LR + SS) corrections across the series (−27 to −29 meV for the cis isomers and −30 to −33 meV for the trans isomers). The remaining larger magnitude of the corrections for the trans isomers increases the cis–trans excitation-energy gap and therefore does not improve the overall agreement with experiment, as the trans excitation energies tend to be more underestimated. Reducing ε_LR_ to ε_*o*_ changes the individual ptLR and ptSS contributions only slightly, with the combined pt­(LR + SS) correction changing by at most 2 meV and therefore not affecting the screening trends discussed above. The corresponding 6-31G* ptLR and ptSS corrections exhibit the same trends and differ from the cc-pVTZ values by at most 1 meV (Table S10). Given the relatively small differences among the calculated excitation energies, cancellation of errors associated with the mean-field electronic-structure treatment, continuum description of the solvent, and perturbative treatment of the non-equilibrium response cannot be excluded. Accordingly, such energy differences should be interpreted with caution.

**Table 7:** Perturbative Linear-Response (ptLR) and State-Specific (ptSS) Corrections and Their Sum (ptLR + SS), in meV, for the SRSH–PCM *S*_1_ Excitation Energies of the AB Derivatives with ε_LR_ = ε_*s*_ and ε_LR_ = ε_*o*_ ^a^

isomer	ptLR		ptSS		pt(LR+SS)	
	ε_s_	ε_o_	ε_s_	ε_o_	ε_s_	ε_o_
1 cis	–20.9	–20.2	–7.9	–8.6	–28.8	–28.8
trans	–15.1	–15.3	–17.9	–16.9	–33.0	–32.2
2 cis	–19.9	–19.3	–7.3	–7.9	–27.2	–27.2
trans	–18.5	–17.6	–12.2	–11.9	–30.7	–29.5
3 cis	–19.8	–19.3	–8.0	–8.5	–27.8	–27.8
trans	–15.1	–14.9	–15.8	–15.2	–30.9	–30.1
4 cis	–20.4	–19.6	–7.1	–7.8	–27.5	–27.4
trans	–18.4	–17.5	–11.9	–11.6	–30.3	–29.1
5 cis	–21.1	–19.9	–6.9	–7.6	–28.0	–27.5
trans	–18.6	–17.3	–12.4	–12.2	–31.0	–29.5
6 cis	–20.3	–19.4	–7.5	–8.1	–27.8	–27.5
trans	–18.8	–17.6	–14.2	–13.4	–33.0	–31.0

a Calculations were performed with α = 0.35 and the cc-pVTZ basis set. Corresponding 6-31G* results are reported in Table S10.

Table 8 summarizes the RMSDs of the SRSH–PCM *S*_1_ excitation energies relative to experiment and the published BSE values. Consistent with the analysis of Table 5, the RMSDs show only a weak dependence on basis set. The dominant effect is the choice of long-range dielectric screening. For comparison with experiment, ε_LR_ = ε_*s*_ generally yields smaller RMSDs than ε_LR_ = ε_*o*_. The RMSD values are small relative to the precision with which the experimental reference energies can be interpreted. We therefore use them primarily to assess the systematic dependence on the choice of long-range dielectric screening. This screening-dependent trend is particularly evident for the cis isomers, for which the RMSDs are below 0.02 eV and are smaller by a factor of two to three than the corresponding ε_LR_ = ε_*o*_ values. The trans isomers show a less uniform behavior due to the highly polarizable 5-trans and 6-trans derivatives, for which the lower-screening ε_LR_ = ε_*o*_ results provide closer agreement with experiment. Excluding these two systems reduces the trans RMSDs obtained with ε_LR_ = ε_*s*_ to 0.015 and 0.027 eV for the 6-31G* and cc-pVTZ basis sets, respectively, comparable to the corresponding cis RMSDs of 0.015 and 0.019 eV.

**Table 8:** RMSD of SRSH–PCM *S*_1_ Excitation Energies Relative to Experimental and Published BSE Values, Evaluated with and without State-Specific (SS) Corrections and Using either ε_o_ or ε_s_ for ε_LR_ ^a^

basis	εLR	experiment			BSE		
set		cis	trans		cis	trans	
6-31G*	ε_*s*_	0.015	0.043	0.032	0.057	0.058	0.057
	ε_*o*_	0.049	0.026	0.039	0.038	0.058	0.049
^b^	ε_*s*_	0.015	0.015	0.015	0.061	0.076	0.067
	ε_*o*_	0.049	0.026	0.041	0.032	0.042	0.036
cc-pVTZ	ε_*s*_	0.019	0.055	0.041	0.077	0.062	0.070
	ε_*o*_	0.036	0.025	0.031	0.039	0.052	0.046
^b^	ε_*s*_	0.019	0.027	0.023	0.084	0.086	0.085
	ε_*o*_	0.036	0.022	0.031	0.039	0.042	0.040

a All results employ α = 0.35 and the appropriate tuning of the range-separation parameter ω (PCM tuning for ε_s_ and gas-phase tuning for ε_o_).

b Experiment: excluding 5-trans and 6-trans. BSE: excluding 2-trans, 3-trans, 6-trans, and 5-cis.

A complementary trend is observed relative to the considered BSE values. Here, ε_LR_ = ε_*o*_ generally yields the smaller RMSDs. The difference is most evident for the cis isomers, where the RMSDs decrease from 0.057 and 0.077 eV with ε_LR_ = ε_*s*_ to 0.038 and 0.039 eV with ε_LR_ = ε_*o*_ for the two basis sets. This behavior is consistent with the optical dielectric screening used in the BSE calculations, whereas the dielectric screening set by ε_*s*_ also accounts for nuclear contributions to the polarization. The trans isomers follow the same trend once the exceptions identified above are excluded, with RMSDs of 0.042 eV for both basis sets with ε_LR_ = ε_*o*_, compared to 0.076 and 0.086 eV with ε_LR_ = ε_*s*_. The resulting trans RMSDs are comparable to those of the cis series for comparisons with both experiment and BSE, indicating that the larger RMSDs of the full trans series are driven by a small number of outliers rather than by a systematic cis–trans difference. Overall, the RMSD analysis is consistent with the trends observed for the individual excitation energies. Static dielectric screening (ε_LR_ = ε_*s*_) yields the closest agreement with experiment, whereas optical dielectric screening (ε_LR_ = ε_*o*_) yields the closest agreement with the considered BSE values.

As a final point, we consider the 1-AB isomers in methanol as the solvent.^76^ Experimental data indicate that the excitation energies of both the cis and trans isomers remain unchanged when moving from acetonitrile to methanol. In agreement with this trend, our calculated excitation energies using the static dielectric constant of methanol, ε_s_ = 32.7, to set ε_LR_ yield the same values as obtained above for acetonitrile. The published BSE excitation energies, however, show small shifts: the cis excitation decreases by 0.04 eV, while the trans excitation increases by 0.06 eV. These shifts are accompanied by corresponding changes in the α parameter used in the BSE calculations, decreasing by 0.01 for the cis isomer and increasing by 0.02 for the trans isomer. In our calculations, the effect of these variations in α can be assessed by adjusting α while keeping the methanol dielectric constant fixed. Specifically, modifying α by −0.02 for the cis isomer and by +0.04 for the trans isomer leads to excitation-energy shifts of −0.02 eV and +0.03 eV, respectively. These changes follow the BSE trends, suggesting that variations in the employed α contribute to the observed solvent-dependent shifts in the BSE excitation energies.

## 4 Conclusions

In this work, we evaluated SRSH–PCM excitation energies for a series of functionalized AB derivatives through comparison with experiment and previously published GW/BSE calculations. SRSH–PCM calculations employing either the static or optical dielectric constant to define the long-range screening yield close agreement with experiment and the published GW–BSE excitation energies, respectively, with mean absolute deviations of 0.03 and 0.04 eV. Comparison with the published GW/BSE results^13^ is particularly valuable because it directly relates the SRSH–PCM mean-field description to a many-body treatment of electronic excitations. The observed trends reflect the distinct physical roles of the two dielectric constants. The optical dielectric constant represents the fast electronic screening in the non-equilibrium GW/BSE embedding treatment, whereas the static dielectric constant also accounts for nuclear contributions to the solvent polarization. The results therefore highlight the importance of accounting for both electronic and nuclear solvent polarization in describing solution-phase excitation energies. An important exception is provided by highly polarizable solutes, for which a reduced effective long-range screening, approximated here by the optical dielectric constant, yields better agreement with experiment. This behavior suggests that strong solute polarization may reduce the effective long-range environmental screening represented by the solvent static dielectric constant, indicating that the solvent static dielectric constant alone may not fully characterize the relevant screening in highly polarizable systems.

Overall, the systematic screening-dependent trends observed across the full set of cis and trans AB derivatives indicate that dielectric screening is a key factor in solution-phase excited-state modeling and demonstrate that SRSH–PCM provides the flexibility to describe different regimes of long-range dielectric screening. By directly relating excitation energies to long-range dielectric screening, SRSH–PCM offers a computationally efficient and physically insightful mean-field framework for investigating environmental screening effects in excited-state calculations. The results also motivate the development of a general procedure for determining the appropriate long-range dielectric screening that accounts for nuclear contributions to the solvent polarization.

## Supplementary Material





## Data Availability

The data required to reproduce the results reported in this work are provided as Supporting Information accompanying this article.

## Notes

*Trans*-1-AB data and the results obtained with the 6-31G* basis set, a list of the attached data files, example Q-Chem input files illustrating the SRSH–PCM setup, and the XYZ coordinates of all investigated molecules (PDF)
Additional data files provided as compressed Q-Chem output archives associated with selected reported results (ZIP)
