# Supplementary material for: Dielectric-Screened Functionals for Cis–Trans Excitation Energies of Solvated Azobenzene Derivatives: Screening Trends in Comparison with Experiment and Published GW/BSE Calculations
Source: J Chem Theory Comput. 2026 Sep 21;22(19):10341–51. doi: 10.1021/acs.jctc.6c01302 (PMC13671178; doi:10.1021/acs.jctc.6c01302)
Supplement: Supplementary file 1 [file ct6c01302_si_001.pdf]

Dielectric-Screened Functionals for Cis–Trans Excitation Energies of Solvated Azobenzene Derivatives: Screening Trends in Comparison with Experiment and Published GW/BSE Calculations

Aswathy Jayachandran and Barry D. Dunietz*

Kent State University, Department of Chemistry and Biochemistry, Kent, OH 44242 USA

Received September 8, 2026; E-mail: bdunietz@kent.edu

This main Supporting Information file includes the supplementary tables referenced in the manuscript, a listing of the output files included with the Supporting Information and corresponding to reported data, and two figures illustrating the SRSH–PCM computational setup. The document concludes with the atomic coordinates of all molecules studied.

SI Table S1. Ground-state properties of trans-1-AB in the gas phase and in acetonitrile solution using the cc-pVTZ basis set. Listed are the IP, EA, HOMO (H), and LUMO (L) energies (eV), along with the tuned range-separation (RS) parameter ω and the error measure J, evaluated for the RSH and SRSH–PCM setups defined by the indicated values of α and $\varepsilon_{\mathrm{LR}}$.

Method	α	ε_{LR}	ω	IP	EA	H	L	J
Gas	0.20	1	176	8.14	0.74	-8.14	-0.75	1.0×10^{-7}
RSH	0.35	1	136	8.22	0.74	-8.21	-0.73	2.0×10^{-7}
	0.45	1	105	8.27	0.74	-8.24	-0.72	2.5×10^{-6}
Condensed	0.20	1	176	6.51	2.72	-8.32	-0.92	8.8×10^{-3}
RSH-PCM	0.35	1	136	6.61	2.74	-8.36	-0.92	8.7×10^{-3}
	0.45	1	105	6.69	2.76	-8.38	-0.91	8.6×10^{-3}
SRSH-PCM	0.20	ε_s	176	6.27	2.72	-6.06	-2.87	9.5×10^{-5}
		ε_o	176	6.41	2.72	-7.31	-1.82	2.2×10^{-3}
	0.35	ε_s	136	6.47	2.73	-6.58	-2.57	4.8×10^{-5}
		ε_o	136	6.56	2.74	-7.55	-1.68	2.9×10^{-3}
	0.45	ε_s	105	6.61	2.75	-6.94	-2.29	4.6×10^{-4}
		ε_o	105	6.65	2.76	-7.72	-1.54	3.6×10^{-3}
(retuned)	0.20	ε_s	67	6.32	2.72	-6.30	-2.70	9.0×10^{-7}
	0.35	ε_s	193	6.42	2.73	-6.40	-2.70	8.0×10^{-7}
	0.45	ε_s	253	6.45	2.74	-6.41	-2.71	2.3×10^{-6}

SI Table S2. Ground-state properties of cis-1-AB in the gas phase and in acetonitrile solution using the 6-31G* basis set. Listed are the IP, EA, HOMO (H), and LUMO (L) energies (eV), along with the tuned range-separation (RS) parameter ω and the error measure J, evaluated for the RSH and SRSH–PCM setups defined by α and $\varepsilon_{\mathrm{LR}}$.

Method	α	ε_{LR}	ω	IP	EA	H	L	J
Gas	0.20	1	201	7.83	-0.10	-7.81	0.11	5.0×10^{-7}
RSH	0.35	1	149	7.89	-0.07	-7.86	0.06	1.3×10^{-6}
	0.45	1	115	7.98	-0.05	-7.93	0.07	4.9×10^{-6}
Condensed	0.20	1	201	6.19	2.02	-8.04	-0.08	9.6×10^{-3}
RSH-PCM	0.35	1	149	6.28	2.08	-8.07	-0.14	9.5×10^{-3}
	0.45	1	115	6.36	2.12	-8.13	-0.14	9.8×10^{-3}
SRSH-PCM	0.20	ε_s	201	5.86	2.02	-5.55	-2.36	3.0×10^{-4}
		ε_o	201	6.05	2.01	-6.91	-1.12	2.0×10^{-3}
	0.35	ε_s	149	6.08	2.06	-6.09	-2.00	2.6×10^{-6}
		ε_o	149	6.20	2.07	-7.17	-0.99	2.8×10^{-3}
	0.45	ε_s	115	6.25	2.11	-6.54	-1.67	3.9×10^{-4}
		ε_o	115	6.31	2.12	-7.41	-0.84	3.9×10^{-3}
(retuned)	0.20	ε_s	21	5.92	2.01	-5.93	-2.02	4.0×10^{-7}
	0.35	ε_s	159	6.07	2.06	-6.05	-2.03	8.0×10^{-7}
	0.45	ε_s	226	6.12	2.10	-6.08	-2.06	3.4×10^{-6}

SI Table S3. Ground-state properties of trans-1-AB in the gas phase and in acetonitrile solution using the 6-31G* basis set. Listed are the IP, EA, HOMO (H), and LUMO (L) energies (eV), along with the tuned range-separation (RS) parameter ω and the error measure J, evaluated for the RSH and SRSH–PCM setups defined by α and $\varepsilon_{\mathrm{LR}}$.

Method	α	ε_{LR}	ω	IP	EA	H	L	J
Gas	0.20	1	191	8.00	0.38	-8.02	-0.38	4.0×10^{-7}
RSH	0.35	1	150	8.09	0.40	-8.11	-0.39	3.0×10^{-7}
	0.45	1	116	8.16	0.42	-8.13	-0.41	1.4×10^{-6}
Condensed	0.20	1	191	6.40	2.41	-8.23	-0.59	9.0×10^{-3}
RSH-PCM	0.35	1	150	6.52	2.46	-8.29	-0.61	8.9×10^{-3}
	0.45	1	116	6.60	2.49	-8.31	-0.63	8.7×10^{-3}
SRSH-PCM	0.20	ε_s	191	6.10	2.39	-5.77	-2.63	2.2×10^{-4}
		ε_o	191	6.27	2.40	-7.13	-1.53	2.0×10^{-3}
	0.35	ε_s	150	6.32	2.43	-6.35	-2.37	3.1×10^{-6}
		ε_o	150	6.44	2.44	-7.41	-1.42	2.7×10^{-3}
	0.45	ε_s	116	6.48	2.47	-6.74	-2.12	2.6×10^{-4}
		ε_o	116	6.55	2.48	-7.60	-1.31	3.4×10^{-3}
(retuned)	0.20	ε_s	34	6.15	2.39	-6.13	-2.36	1.2×10^{-6}
	0.35	ε_s	163	6.31	2.42	-6.30	-2.41	2.0×10^{-7}
	0.45	ε_s	219	6.36	2.46	-6.34	-2.43	1.3×10^{-6}

SI Table S4. Ground-state properties of the AB derivatives solvated in acetonitrile, calculated at the SRSH–PCM level with $\varepsilon_{\rm LR} = \varepsilon_s$, $\alpha = 0.35$, and the PCM-tuned RS parameter using the 6-31G* basis set. Listed are the OP, RP, HOMO (H), and LUMO (L) energies (eV), along with the tuned RS parameter ω and the error measure J.

AB	ω	OP	RP	H	L	J
1 cis	159	6.07	2.06	-6.05	-2.03	8.0×10^{-7}
trans	163	6.31	2.42	-6.30	-2.41	2.0×10^{-7}
2 cis	141	6.64	2.46	-6.64	-2.42	4.0×10^{-7}
trans	150	6.43	2.62	-6.43	-2.60	0.0×10^{-7}
3 cis	151	6.37	2.25	-6.35	-2.22	8.0×10^{-7}
trans	157	6.41	2.53	-6.41	-2.51	0.0×10^{-7}
4 cis	136	6.65	2.51	-6.63	-2.46	1.0×10^{-6}
trans	143	6.45	2.62	-6.45	-2.59	1.0×10^{-7}
5 cis	143	6.70	2.68	-6.68	-2.65	7.0×10^{-7}
trans	151	6.51	2.87	-6.51	-2.85	0.0×10^{-7}
6 cis	143	7.08	3.04	-7.07	-3.03	0.0×10^{-7}
trans	150	6.86	3.29	-6.87	-3.29	4.0×10^{-7}

SI Table S5. Ground-state properties of the AB derivatives in the gas phase, calculated at the RSH level with $\alpha = 0.35$ using the cc-pVTZ basis set. Listed are the IP, EA, HOMO (H), and LUMO (L) energies (eV), along with the tuned RS parameter ω and the error measure J.

AB	ω	IP	EA	H	L	J
1 cis	140	8.02	0.24	-7.98	-0.22	2.7×10^{-6}
trans	136	8.22	0.74	-8.21	-0.73	2.0×10^{-7}
2 cis	141	8.58	0.66	-8.56	-0.64	1.9×10^{-6}
trans	133	8.31	0.99	-8.33	-1.01	1.1×10^{-6}
3 cis	140	8.32	0.44	-8.28	-0.42	3.0×10^{-6}
trans	134	8.31	0.86	-8.32	-0.87	3.0×10^{-7}
4 cis	142	8.71	0.82	-8.67	-0.79	3.2×10^{-6}
trans	135	8.43	1.10	-8.44	-1.10	3.0×10^{-7}
5 cis	128	8.66	1.13	-8.61	-1.09	4.2×10^{-6}
trans	122	8.41	1.46	-8.40	-1.46	2.0×10^{-7}
6 cis	131	9.31	1.79	-9.29	-1.80	5.0×10^{-7}
trans	123	9.05	2.14	-9.07	-2.18	1.4×10^{-6}

SI Table S6. Frontier orbital energies (eV)—HOMO (H) and LUMO (L)—of the AB derivatives, calculated at the SRSH–PCM level with $\varepsilon_{\rm LR} = \varepsilon_o$, $\alpha = 0.35$, and the gas-phase-tuned ω using the 6-31G* basis set.

AB	ω	H	L
1 cis	149	-7.17	-0.99
trans	150	-7.41	-1.42
2 cis	153	-7.69	-1.42
trans	144	-7.50	-1.65
3 cis	153	-7.44	-1.19
trans	146	-7.50	-1.54
4 cis	154	-7.67	-1.47
trans	148	-7.49	-1.66
5 cis	141	-7.71	-1.68
trans	131	-7.53	-1.94
6 cis	143	-8.11	-2.07
trans	133	-7.91	-2.39

SI Table S7. S_1 excitation energies (eV) of the AB derivatives calculated at the SRSH–PCM level with $\alpha = 0.35$ and $\varepsilon_{\mathrm{LR}}$ set to either ε_s or ε_o, using the 6-31G* basis set. For $\varepsilon_{\mathrm{LR}} = \varepsilon_s$, ω was tuned in the PCM environment; for $\varepsilon_{\mathrm{LR}} = \varepsilon_o$, the gas-phase-tuned ω was used.

AB	$\varepsilon_{LR} =$		Exp	BSE
isomer	ε_s	ε_o		
1 cis	2.90	2.94	2.88	2.95
trans	2.78	2.82	2.79	2.83
2 cis	3.00	3.03	2.99	3.03
trans	2.70	2.73	2.72	2.67
3 cis	2.96	3.00	2.96	3.01
trans	2.75	2.79	2.75	2.72
4 cis	2.99	3.02	2.99	3.02
trans	2.74	2.77	2.76	2.81
5 cis	2.97	3.01	2.94	2.95
trans	2.65	2.69	2.71	2.75
6 cis	2.95	2.99	2.95	3.06
trans	2.56	2.61	2.64	2.53

SI Table S8. SRSH–PCM S_1 excitation energies (eV) of the AB derivatives calculated with $\varepsilon_{\mathrm{LR}} = \varepsilon_s$, $\alpha = 0.35$, and the gas-phase-tuned ω using the 6-31G* basis set.

AB	S1
1 cis	2.90
trans	2.79
2 cis	2.99
trans	2.70
3 cis	2.96
trans	2.76
4 cis	2.98
trans	2.74
5 cis	2.97
trans	2.66
6 cis	2.95
trans	2.57

SI Table S9. SRSH–PCM S_1 excitation energies (eV) of the AB derivatives calculated with $\varepsilon_{\mathrm{LR}} = \varepsilon_o$, $\alpha = 0.35$, and the PCM-tuned ω using the 6-31G* basis set.

AB	S1
1 cis	2.94
trans	2.82
2 cis	3.03
trans	2.73
3 cis	3.00
trans	2.79
4 cis	3.02
trans	2.77
5 cis	3.01
trans	2.69
6 cis	2.99
trans	2.62

SI Table S10. Perturbative linear-response (ptLR) and state-specific (ptSS) corrections and their sum (ptLR+SS) in meV for the SRSH–PCM S_1 excitation energies of the AB derivatives, evaluated for two choices of the long-range dielectric screening parameter, $\varepsilon_{\rm LR} = \varepsilon_s$ and $\varepsilon_{\rm LR} = \varepsilon_o$. Calculations were performed with $\alpha = 0.35$ and the 6-31G* basis set.

AB	ptLR		ptSS		pt(LR+SS)	
isomer	ε_s	ε_o	ε_s	ε_o	ε_s	ε_o
1 cis	-21.9	-21.4	-8.9	-9.6	-30.8	-31.0
trans	-14.5	-14.6	-17.6	-16.9	-32.1	-31.5
2 cis	-21.0	-20.6	-8.1	-8.6	-29.1	-29.2
trans	-17.7	-17.0	-12.4	-12.3	-30.1	-29.3
3 cis	-20.9	-20.5	-9.0	-9.5	-29.9	-30.0
trans	-14.5	-14.4	-15.7	-15.3	-30.2	-29.7
4 cis	-21.6	-21.1	-7.9	-8.5	-29.5	-29.6
trans	-17.7	-17.0	-12.1	-11.9	-29.8	-28.9
5 cis	-21.8	-21.1	-7.7	-8.4	-29.5	-29.5
trans	-17.6	-16.6	-12.7	-12.5	-30.3	-29.1
6 cis	-21.3	-20.7	-8.2	-8.8	-29.5	-29.5
trans	-18.3	-17.2	-14.3	-13.7	-32.6	-30.9

Computational Input Setup and Output Files

- Table1_SubPC_0.2tune_631gd.tar.gz: Neutral, anion and cation output files of SubPC molecule at gas phase and condensed phase with α=0.2 and 6-31G* basis set (Table 1).

- Table2_1abcis_0.2tune_ccpvtz.tar.gz : Neutral, anion and cation output files of cis-1-ab- molecule at gas phase and condensed phase with α=0.2 and cc-pVTZ basis set (Table 2).

- SItableS1_1abtrans_0.2tune_ccpvtz.tar.gz : Neutral, anion and cation output files of trans-1-ab molecule at gas phase and condensed phase with α=0.2 and cc-pVTZ basis set (Table S1).

- SItableS2_1abcis_0.2tune_631gd.tar.gz : Neutral, anion and cation output files of cis-1-ab molecule at gas phase and condensed phase with α=0.2 and 6-31G* basis set (Table S2).

- SItableS3_1abtrans_0.2tune_631gd.tar.gz : Neutral, anion and cation output files of trans-1-ab molecule at gas phase and condensed phase with α=0.2 and 6-31G* basis set (Table S3).

- Table5_es-ccpvtz.tar.gz : Excited state energies of all AB isomers at $\varepsilon_{\mathrm{LR}} = \varepsilon_s$ and cc-pVTZ basis set (Table 5).

- Table5_eo-ccpvtz.tar.gz : Excited state energies of all AB isomers at $\varepsilon_{\mathrm{LR}} = \varepsilon_o$ and cc-pVTZ basis set (Table 5).

- SITableS7_es-631gd.tar.gz : Excited state energies of all AB isomers at $\varepsilon_{\mathrm{LR}} = \varepsilon_s$ and 6-31G* basis set (Table S7).

- SITableS7_eo-631gd.tar.gz : Excited state energies of all AB isomers at $\varepsilon_{\mathrm{LR}} = \varepsilon_o$ and 6-31G* basis set (Table S7).

$$E_{XC}^{\mathrm{RSH}} = \alpha E_{F_X}^{\mathrm{SR}} + (1-\alpha)E_{DF_X}^{\mathrm{SR}} + E_{F_X}^{\mathrm{LR}} + E_{DF_C}$$

```
$molecule
read                      neutral.txt
$end

$XC_FUNCTIONAL
X HF     0.20     (α)
X wPBE   0.80     (1- α )
C PBE    1.00
$end

$rem
jobtype                   sp
exchange                  gen
correlation               none
lrc_dft                   true
basis                     cc-pvtz
omega                     184
scf_convergence           8
geom_opt_maxcyc           500
max_scf_cycles            5000
mem_static                4000
mem_total                 48000
print_general_basis       true
print_orbitals            10
molden_format             true
$end
```

SI Figure 1. Sample Q-Chem input for an RSH calculation in the gas phase using the cc-pVTZ basis set with $\alpha = 0.20$.

$$E_{XC}^{\mathrm{SRSH}} = \alpha E_{F_x}^{\mathrm{SR}} + (1-\alpha)E_{DF_x}^{\mathrm{SR}} + (\alpha + \beta)E_{F_x}^{\mathrm{LR}} + (1 - \alpha - \beta)E_{DF_x}^{\mathrm{LR}} + E_{DF_C}$$

```
$molecule
read                      neutral.txt
$end

$XC_FUNCTIONAL
X HF   0.200       (α)
X PBE  0.973       (1-(1/Ɛ))
X wPBE -0.173      (1/Ɛ - α)
C PBE  1.00
$end

$rem
jobtype                   sp
exchange                  gen
correlation               none
lrc_dft                   true
hfk_sr_coef               20000000     (α * 10^8)
hfk_lr_coef               2700000      (1/Ɛ * 10^8)
basis                     cc-pVTZ
omega                     50
omega2                    50
scf_convergence           8
geom_opt_maxcyc           500
max_scf_cycles            5000
mem_static                10000
mem_total                 100000
print_orbitals            10
molden_format             true
solvent_method            pcm
$end

$pcm
Theory      cpcm
Method      SWIG
Solver      Inversion
HeavyPoints 194
HPoints     194
Radii       bondi
vdwScale    1.2
$end

$solvent
Dielectric 37.5
$end
```

SI Figure 2. Sample Q-Chem input for an SRSH–PCM calculation using the cc-pVTZ basis set with $\alpha = 0.20$ and $\varepsilon = 37.5$. The exchange-functional weights are set to PBE $= 1 - 1/\varepsilon$ and wPBE $= 1/\varepsilon - \alpha$. The `hfk_sr_coef` and `hfk_lr_coef` values are specified by multiplying the corresponding weights by 10^8.

SI Table S11. Atomic coordinates of optimized SubPC molecule (Å)

H	-3.2383637075434892	-5.0887952885333414	2.2448521097403757
C	-3.5817177639808566	-4.9111124296168169	1.2303877902040465
C	-4.7742718788643943	-5.4552686976910811	0.7704031153216437
H	-5.3783412672012290	-6.0625128652358011	1.4377904154269221
C	-5.2146836824035363	-5.2414043161017743	-0.5492110499164553
H	-6.1499750437957603	-5.6856300847074710	-0.8763066871136902
C	-4.4728486269394931	-4.4777811425498379	-1.4411963549507842
H	-4.8060233101415966	-4.3242093540307591	-2.4629481183785722
C	-3.2849633038199970	-3.9056552070051578	-0.9833716381788576
C	-2.2539539006484315	-3.1353139335327471	-1.6614657123189847
C	-2.8393930526221842	-4.1210525744944517	0.3506289464943090
C	-1.5388669740106433	-3.4797506310554724	0.4768088695491915
N	-0.5461442691820796	-3.6847022067773274	1.3494315428130559
N	-1.3502102256322444	-2.8092264606986785	-0.6950530808982904
N	-1.9908032259636537	-2.9892109433032519	-2.9645959380748295
C	0.6934871509536754	-3.3559447158561748	0.9696784301724011
C	-0.7413600937561582	-2.6644328677109299	-3.3138081160748682
N	0.9452730806225960	-2.6831639033300467	-0.1883799473730323
C	1.9865791240408963	-3.8502659427866393	1.4187073011459268
C	2.9269950605140891	-3.5585955540817422	0.3914224261357716
C	2.3680858288211271	-4.5717275169212996	2.5510932299374889
C	3.6944544938476236	-4.9720890584796811	2.6524501130556644
C	4.6242936692502052	-4.6844532228205917	1.6359644900694956
C	4.2511161914072684	-3.9881497642134192	0.4932553167496933
C	2.2010256809586739	-2.8874085176526050	-0.6766995554171585
N	2.4957346427125953	-2.7387759342097482	-1.9727533135629240
C	1.4818712155287852	-2.5394756310445583	-2.8219703822684270
N	0.2058682949145901	-2.3248433222203038	-2.3945231974214396
B	-0.1221261592578121	-2.0391464876990719	-0.9819778396972884
Cl	-0.3004430777706568	-0.1938236510812711	-0.6206923847975855
C	-0.0147500908629507	-2.8875757233475756	-4.5546768460853517
C	1.3724759659127881	-2.8087770232728957	-4.2477548029492986
C	-0.4430301872480531	-3.2244187743656552	-5.8398632447236016
C	2.3350975902850242	-3.0649037495982188	-5.2255100757123865
C	0.5243348544596503	-3.4532790218704328	-6.8101234123479646
C	1.8964787204583142	-3.3742271167916944	-6.5066303387255529
H	1.6487715646076124	-4.8057705032190388	3.3296116718403197
H	4.0220912171596721	-5.5207572258876167	3.5303778566022554
H	5.6520211704391317	-5.0168853173277812	1.7477400758435986
H	4.9674092434390023	-3.7776916814942179	-0.2947861904186639
H	-1.5009665117683422	-3.3002662373570972	-6.0716501860554120
H	3.3939192400773281	-3.0167837367921919	-4.9904020890771532
H	0.2196388306734977	-3.7030840480943557	-7.8220456926185529
H	2.6246735223295521	-3.5643476151393076	-7.2893575059449986

SI Table S12. Atomic coordinates of optimized cis-1-AB (Å)

C	-2.9769297105647348	1.7620441785052707	-0.2566434431148659
C	-1.6046689707053416	1.6741368105201238	-0.4280295795522157
C	-0.8008107463796313	2.7387206990947885	-0.0375693117138992
C	-1.3557355230799926	3.8665221878093008	0.5510815217876636
C	-2.7312994516478182	3.9572906009254325	0.6906666047392047
C	-3.5439675033732372	2.9057541828628621	0.2889153131672925
H	-3.6060528301059263	0.9338390841499050	-0.5530282057411782
H	-1.1606791740669888	0.7848738506164240	-0.8530759207149738
H	-0.7080908925764586	4.6687747860142146	0.8780235502982364
H	-3.1674305119317765	4.8463094893545238	1.1251365075327784
H	-4.6163170729374512	2.9731394509208817	0.4117732555819993
N	0.6310083112255160	2.6575557969013888	-0.0912203754063650
N	1.2409786624319987	2.4291686887592725	-1.1388909178184192
C	0.5828008574095760	2.3581822394653691	-2.4121437628709090
C	0.8208073735350883	1.2352282287059577	-3.1924523803681368
C	-0.1501352453308615	3.4274311319266739	-2.9132529188062506
C	0.2653340539435944	1.1537186333151523	-4.4593559209351303
H	1.4248541157478407	0.4294052510287267	-2.7978348497562120
C	-0.6760981994865930	3.3488694181368257	-4.1926713163731399
H	-0.2970869034055819	4.3133090737732669	-2.3116176995782403
C	-0.4828390216372752	2.2095784650939967	-4.9621607738809672
H	0.4280261223046238	0.2693611179934323	-5.0599966653893018
H	-1.2390702769251019	4.1828565049097532	-4.5889245858555912
H	-0.9055974624434668	2.1509301292165208	-5.9557281252313610

SI Table S13. Atomic coordinates of optimized trans-1-AB (Å)

C	-2.7089540268675303	2.1992312106015208	-1.1789608555365376
C	-1.3261491822904481	2.1963871603090839	-1.2098307428555419
C	-0.6150735210734616	2.5594245675495153	-0.0676037178900754
C	-1.2892238887627983	2.9052387097400203	1.0974200848434568
C	-2.6753137779533662	2.9186551791337045	1.1174448188620874
C	-3.3860404061967202	2.5650467310786298	-0.0200619291094374
H	-3.2651291412970953	1.9094832638230517	-2.0601942070387285
H	-0.7935467213121938	1.9052179366029360	-2.1033051744831668
H	-0.7151981279966322	3.1690871013022432	1.9755357135871554
H	-3.1983915337719595	3.2004080741755878	2.0208599232607956
H	-4.4676442027352419	2.5650894253138365	-0.0046147596963969
N	0.8039667015354495	2.5786209354049761	0.0125432094230057
N	1.3931431677060335	2.5768265393004275	-1.0761642650708441
C	2.8122429116370502	2.5580337537695463	-0.9965939593362771
C	3.4857717875869869	2.8999246816912403	-2.1631445064385000
C	3.5239641382513325	2.1989482080454197	0.1464839398013294
C	4.8718485369056701	2.9133851490008511	-2.1839594491376819
H	2.9112343067686015	3.1607228097327953	-3.0418358712386673
C	4.9067564139173445	2.2018183994482134	0.1148722734337184
H	2.9918359633142457	1.9108046733217334	1.0412193374435914
C	5.5831988324791180	2.5637135694667093	-1.0456294947470866
H	5.3944538986237918	3.1920021980030664	-3.0886198661623641
H	5.4634525827593485	1.9151431887907695	0.9967818449214602
H	6.6647952887724653	2.5637865343940498	-1.0616423468352565

SI Table S14. Atomic coordinates of optimized cis-2-AB (Å)

C	-2.9962174478563806	1.7098123607103919	-0.2075542145555491
C	-1.6298075186420187	1.6618291028584138	-0.3833770696844165
C	-0.8020286498449961	2.7304312011871525	-0.0586655749762857
C	-1.4087298971640183	3.8508544232247841	0.4958906421833104
C	-2.7707730486390680	3.9443573442407263	0.6721333983648907
C	-3.5611743474672739	2.8619288348816192	0.3147229606300305
H	-3.5959432123622825	0.8518992283578453	-0.4737286876647343
F	-1.0720460620728263	0.5403958571742048	-0.8526057180264422
F	-0.6256678995758177	4.8782633532398689	0.8361886960557380
H	-3.1946443106972917	4.8481823001828808	1.0840294041128404
H	-4.6316253048738742	2.9162894940954174	0.4511018439387842
N	0.6254678694360718	2.6633594727778815	-0.0970936522856192
N	1.2357382559084471	2.4398748326353696	-1.1448649323919715
C	0.5651412241104442	2.3742925495243750	-2.4055266059736367
C	0.7364936119166205	1.2495966555344800	-3.2032121226961991
C	-0.1140468544903115	3.4482805527956120	-2.9684498743107408
C	0.2173909101421754	1.1576744900169702	-4.4748890528110064
F	1.4064277575809914	0.2160683558884040	-2.6856033341515504
C	-0.6326180835548159	3.4031754991441132	-4.2448140379466519
F	-0.2376178517841749	4.5709961019857568	-2.2516637972152664
C	-0.4692926214396297	2.2469339948935008	-4.9906598252808951
H	0.3528035321877082	0.2492468120287434	-5.0428205972332618
H	-1.1460215806050300	4.2676912163606993	-4.6390133200113217
H	-0.8801784702126441	2.1955659662608076	-5.9885245280700543

SI Table S15. Atomic coordinates of optimized trans-2-AB (Å)

C	-2.7415762770124639	1.9578636782477394	-1.0805984627090139
C	-1.3635808262734372	1.9958145582446012	-1.1132998353173931
C	-0.6111370871794000	2.5936298358668424	-0.0969604843393658
C	-1.3328121582549501	3.1392247113487013	0.9670716493694232
C	-2.7084585718416987	3.1356908263838852	1.0225704641140421
C	-3.4086779338540705	2.5381769555434457	-0.0136383190537638
H	-3.2718555552439437	1.4730190416293976	-1.8870173834187083
F	-0.7441118986250782	1.3969467460197162	-2.1300898447424519
F	-0.6492617445585019	3.7068094911835283	1.9629143139410508
H	-3.2088441007501642	3.5897455879440217	1.8647905442779882
H	-4.4888647557263273	2.5186966005125258	0.0134430988277509
N	0.7930085483059691	2.6264453199751099	0.0083957402855373
N	1.4040569015549555	2.6243274997393184	-1.0726291992522159
C	2.8082838827196466	2.5921252150701073	-0.9676103523726765
C	3.5295031577836817	3.1341607710344137	-2.0337496647489708
C	3.5612426061809601	1.9979534841179909	0.0505018908601999
C	4.9051300733022538	3.1307672562372222	-2.0897119130459041
F	2.8454700686469208	3.6981810880909185	-3.0312918468709875
C	4.9392356770178454	1.9601777754699552	0.0174408305991573
F	2.9424118070289360	1.4023303202951662	1.0695838709116803
C	5.6058598644615092	2.5369771553470497	-1.0517152973932873
H	5.4050675088108484	3.5821275141652045	-2.9336427239539473
H	5.4698496801254173	1.4781390774913723	0.8253177584642051
H	6.6860411333810825	2.5176594900417735	-1.0790948344323308

SI Table S16. Atomic coordinates of optimized cis-3-AB (Å)

C	-3.0231338619015546	1.7169341215981579	-0.1723011200310945
C	-1.6570715819495980	1.6370128397601820	-0.3412319535058769
C	-0.8102982213635335	2.7011288069980401	-0.0619236259431388
C	-1.3976184208584039	3.8560559171275122	0.4341924133780750
C	-2.7578260479489107	3.9831556056195687	0.6071900979367960
C	-3.5684025120715259	2.9003539620536198	0.2995481199539258
H	-3.6390299920429374	0.8613936450352689	-0.4075371365425943
F	-1.1167351478125318	0.4921750392929838	-0.7766734788809072
F	-0.5944415330711597	4.8905155847077477	0.7134199077868705
H	-3.1649219618334850	4.9123425138218249	0.9778086059067520
H	-4.6376661278803990	2.9801905243428499	0.4326315063537770
N	0.6212684315193913	2.5865535662913866	-0.0826745316875935
N	1.2379746683476969	2.3692473459383985	-1.1277590726352691
C	0.5940064631903345	2.3457025634085333	-2.4048901741001973
C	0.8366846705504956	1.2457214421108764	-3.2167602339117054
C	-0.1438118001599644	3.4273194885347178	-2.8713839552952534
C	0.2780496507547408	1.1983945595734642	-4.4829688353031019
H	1.4432797702142008	0.4316518013466345	-2.8445948714887099
C	-0.6733331986042245	3.3820740640448270	-4.1510716852618561
H	-0.2811705896126919	4.3021902287448199	-2.2519716334589144
C	-0.4771048861789237	2.2654068205118061	-4.9515188223460385
H	0.4440182938524677	0.3336221275701495	-5.1103295985021475
H	-1.2395794200909767	4.2247953160513196	-4.5229101585661811
H	-0.9021066450484598	2.2330621155152963	-5.9452897638556470

SI Table S17. Atomic coordinates of optimized trans-3-AB (Å)

C	-2.7600805992753878	2.1048356627739389	-1.1465810877704392
C	-1.3811006059851960	2.0960280332064971	-1.1796747009412678
C	-0.6069242106899024	2.5255179420811578	-0.0987220706775544
C	-1.3070963432649789	2.9504108893818297	1.0306873280512725
C	-2.6820618602649269	2.9928504753622289	1.0918226665713915
C	-3.4057326053175112	2.5649626083133183	-0.0102519138172771
H	-3.3077182190644439	1.7486494561239989	-2.0068004632249323
F	-0.7811103011878489	1.6113389372194658	-2.2679893078388256
F	-0.6005370503898206	3.3553322447209859	2.0896914333577783
H	-3.1641716255719019	3.3551909736082148	1.9876306138523698
H	-4.4858053378793912	2.5842853513444290	0.0192788953020081
N	0.8001916274718985	2.4853069687440077	-0.0104442380642783
N	1.4013225807672920	2.7008711386398874	-1.0730524400190062
C	2.8173775545616015	2.6200783488665613	-0.9891969980382820
C	3.5021666927744652	3.0129767531980898	-2.1326711302037991
C	3.5156374926926177	2.1754517174944499	0.1326051836186213
C	4.8878682900130164	2.9768024635062531	-2.1569285021118998
H	2.9366127000453099	3.3466695257640739	-2.9921358295917058
C	4.8969511688186476	2.1374353344414012	0.0998175137109129
H	2.9748056612923808	1.8598546930903483	1.0125386420032336
C	5.5854142810530885	2.5393141048998520	-1.0411620806468638
H	5.4199874211862999	3.2882121567719209	-3.0450630920597894
H	5.4435204754380146	1.7894480743951067	0.9656605643202477
H	6.6664628127766710	2.5051661460520060	-1.0580789857819228

SI Table S18. Atomic coordinates of optimized cis-4-AB (Å)

C	-2.9932039821928562	1.7204946633163183	-0.2048976818119992
C	-1.6275244813846714	1.6781286491495175	-0.3864576263422588
C	-0.7923995322205837	2.7380465859672287	-0.0513081214487794
C	-1.3939585815836861	3.8516339870137073	0.5236304194392821
C	-2.7532519754000702	3.9554670029037342	0.7125330969255789
C	-3.5246633475278046	2.8728489428052213	0.3367179764579925
H	-3.6157138658747470	0.8800315415050600	-0.4717202132547544
F	-1.0838129011418025	0.5611275402413376	-0.8710152831090497
F	-0.6120455408488037	4.8709996492942560	0.8737322221214706
H	-3.1913326482032751	4.8459887220398983	1.1365847233050344
F	-4.8454593274572355	2.9421846095568260	0.5123765257570609
N	0.6343912808316116	2.6682800855701538	-0.0922281300758085
N	1.2434620819975917	2.4349879300303510	-1.1391683952967009
C	0.5745470165047132	2.3665662858342631	-2.4002474137226644
C	0.7665870065673523	1.2483232855617632	-3.2031601118495567
C	-0.1166454716868768	3.4322354299207283	-2.9652985215425027
C	0.2608174988614912	1.1461219226973467	-4.4791544861634707
F	1.4445911078702212	0.2228660950525730	-2.6912225071204339
C	-0.6297699909582944	3.3925548314545804	-4.2439078294142423
F	-0.2593770621158421	4.5517740625591543	-2.2546811574494088
C	-0.4322710672097294	2.2356995273169908	-4.9696490561376292
H	0.4008536379378490	0.2514921286943690	-5.0663579641767216
H	-1.1536062554094038	4.2405537121465784	-4.6579221212328363
F	-0.9291835993551899	2.1685928093680387	-6.2061783438576041

SI Table S19. Atomic coordinates of optimized trans-4-AB (Å)

C	-2.7494411311598150	2.0137767642616295	-1.1166464584610496
C	-1.3718738739208378	2.0510592442616016	-1.1437759487673635
C	-0.6114390371544817	2.5908950082285216	-0.0998759211741009
C	-1.3305400549922528	3.0807653738465595	0.9937211053001384
C	-2.7052823552956311	3.0836989736855602	1.0610308021411599
C	-3.3851669319799971	2.5433060085238157	-0.0118020009012603
H	-3.3033538945786907	1.5772357640122932	-1.9337783853831267
F	-0.7655592448850573	1.5027909584393553	-2.1910724955046357
F	-0.6492152150633239	3.5902860278423452	2.0163709401734251
H	-3.2206682173902852	3.4911152099066749	1.9170960591476063
F	-4.7176820629689216	2.5254313990191810	0.0231060628821682
N	0.7905113189636850	2.6208491313243258	0.0068462727466272
N	1.4066233568444222	2.6187685539863788	-1.0722524652697167
C	2.8085782212980055	2.5895151785580990	-0.9653041405089580
C	3.5276981747799518	3.0757513508320145	-2.0605069319443841
C	3.5690374989783460	2.0534404474537009	0.0805087233487894
C	4.9024432201882604	3.0787701937945209	-2.1276814635964567
F	2.8463626176269181	3.5816019454599592	-3.0849767066247655
C	4.9466193515431165	2.0163645124610468	0.0536496950454317
F	2.9627465202757182	1.5086033263777152	1.1296192230305671
C	5.5823542334508831	2.5422361040944605	-1.0529310207231595
H	5.4178186265065804	3.4834380780232213	-2.9850567362666216
H	5.5005321032193191	1.5827298104091114	0.8723267301101338
F	6.9148767757140632	2.5245606351978855	-1.0876349388004543

SI Table S20. Atomic coordinates of optimized cis-5-AB (Å)

C	-2.9473573563186877	1.7005637837889258	-0.1625324289169661
C	-1.5817177305409598	1.6639892892143080	-0.3446339470035215
C	-0.7568364767521792	2.7369616321907824	-0.0307567763381831
C	-1.3673574628895002	3.8569217458005900	0.5195619265748556
C	-2.7293240798757710	3.9514553060111277	0.7027502306624388
C	-3.5030780909188390	2.8569750332361852	0.3542686013447300
H	-3.5440688055745779	0.8390995430355388	-0.4201239198014622
F	-1.0286646386851539	0.5436924510744987	-0.8109659050577750
F	-0.5945985032465295	4.8895204029243109	0.8509794053332991
H	-3.1558167176394170	4.8561002886903326	1.1087262956551309
Br	-5.3773764459483573	2.9307843781556975	0.5962805203765029
N	0.6693012080917152	2.6693905028763227	-0.0735019270945137
N	1.2732966567501529	2.4330968787524863	-1.1216694861543999
C	0.5987734798963860	2.3660985132133168	-2.3793574019326367
C	0.7646648736915128	1.2419378327570436	-3.1783388800175256
C	-0.0707895477773258	3.4435665880484314	-2.9457427277315231
C	0.2588934697319462	1.1521611098242197	-4.4559652021270999
F	1.4197259567675546	0.2022963159344160	-2.6649014241056563
C	-0.5772774267030162	3.4126307696992364	-4.2276991387383172
F	-0.1973217911834732	4.5642645443878189	-2.2335748531522719
C	-0.4082483842922132	2.2541647058833649	-4.9644623433584449
H	0.3927067515055844	0.2454869187239777	-5.0255315900168958
H	-1.0772126594985967	4.2822942764516583	-4.6255959606320163
Br	-1.0892862785902211	2.1835471893254677	-6.7262130677677243

SI Table S21. Atomic coordinates of optimized trans-5-AB (Å)

C	-2.7508234886069163	2.0251351041857557	-1.1125156029907848
C	-1.3723753069287710	2.0555598856698092	-1.1422509707519761
C	-0.6099023191943092	2.5860383116107948	-0.0965605898575088
C	-1.3261626908284854	3.0715604397418330	0.9995941786434984
C	-2.7018161207431124	3.0788645667109393	1.0650351201966610
C	-3.3977530372271700	2.5484672630195848	-0.0069877240275087
H	-3.2877098219355636	1.5933842219114949	-1.9433011619067784
F	-0.7701501351040940	1.5138215622840088	-2.1952671750117041
F	-0.6435845799987591	3.5721328410473658	2.0259298073738070
H	-3.1959947291631372	3.4858412069997327	1.9337387104576089
Br	-5.2853032331842593	2.5268061132981416	0.0431878839481588
N	0.7924396921039680	2.6135509721281394	0.0077811545060065
N	1.4046980539512235	2.6115069510698530	-1.0731901602010296
C	2.8070426080276367	2.5846780947583139	-0.9686322746194339
C	3.5233452322185328	3.0665348171885776	-2.0663720694820675
C	3.5695180212329491	2.0579803595214372	0.0789614276506326
C	4.8990025345681065	3.0739419369698071	-2.1316593966787734
F	2.8407790213090278	3.5634036726821439	-3.0945201751464544
C	4.9479772922851222	2.0277154792288896	0.0494808922615974
F	2.9672967478163113	1.5197283094540792	1.1337733934646546
C	5.5949463922762366	2.5473911706665153	-1.0577440084055820
H	5.3931737826747232	3.4781509306171605	-3.0016594431396406
H	5.4848328079109940	1.5988718199446397	0.8817874740329977
Br	7.4825032765397816	2.5259239692910485	-1.1076288903163849

SI Table S22. Atomic coordinates of optimized cis-6-AB (Å)

C	-2.9388382871009791	1.6806800561034589	-0.1556738709588504
C	-1.5755457993895523	1.6443198749039043	-0.3409360395280719
C	-0.7573914251773054	2.7264521224284817	-0.0376122384749174
C	-1.3613482315792196	3.8542362773548673	0.5062745313314952
C	-2.7197516474293959	3.9436692588319500	0.6963046064570538
C	-3.5016516778941997	2.8430457467309309	0.3581698172070298
H	-3.5400465806612313	0.8198984141113003	-0.4066322262513336
F	-1.0155029606397958	0.5288966551341868	-0.8040370883955189
F	-0.5861277117152305	4.8861365074952765	0.8225048488365011
H	-3.1514212120965790	4.8467445820591673	1.1003346826973848
C	-4.9197112769500029	2.9078740754158612	0.5474362741150272
N	0.6685916294132283	2.6668511843758869	-0.0877006863317356
N	1.2716359143612492	2.4530883204323417	-1.1391924424620383
C	0.5940297057557304	2.3810720244684362	-2.3939978712782155
C	0.7587431158485204	1.2465552785999949	-3.1799567526286401
C	-0.0828194844580849	3.4573587573929947	-2.9556404112941919
C	0.2362252528139287	1.1457013104881721	-4.4472163987778419
F	1.4228051896284279	0.2189776758262832	-2.6610675498874099
C	-0.6087268971808536	3.4111011219690774	-4.2266724679530689
F	-0.2034145909606022	4.5763481895863167	-2.2442008898481087
C	-0.4491532874542405	2.2422720805370360	-4.9618851864392317
H	0.3609830345065504	0.2357836463614225	-5.0142317517285120
H	-1.1246088523496973	4.2704736652148343	-4.6273836018591323
C	-0.9990466905130034	2.1650327573881305	-6.2818645585907023
N	-1.4400935753534736	2.1018321610595767	-7.3400867607792053
N	-6.0566336534241980	2.9597982557300733	0.6989240328222158

SI Table S23. Atomic coordinates of optimized trans-6-AB (Å)

C	-2.7311731434792104	1.9364158349585450	-0.9857114108408097
C	-1.3545025664040009	1.9682480299944236	-1.0148813614711900
C	-0.6061309905763086	2.6111924676764398	-0.0250377917241045
C	-1.3176119030389719	3.2120309137966707	1.0144954032238751
C	-2.6914972346645505	3.2202031230032486	1.0735136902713009
C	-3.3897986901741550	2.5743568374037138	0.0590407849979076
H	-3.2701266827824838	1.4205521711418110	-1.7659248196218125
F	-0.7374959858077486	1.3244561888874864	-1.9970705481241231
F	-0.6322791262234312	3.8185748158861812	1.9767785302678880
H	-3.1960492479817972	3.7128271303809601	1.8905357729038086
C	-4.8217160744056065	2.5593870821798772	0.0949167783486923
N	0.7993403982353510	2.6327827929642020	0.0752384511834017
N	1.3986695290528022	2.6276857232202855	-1.0104385271249163
C	2.8034466768511841	2.5784520170452212	-0.9110312033250121
C	3.5258954271947318	3.1690911709672189	-1.9487636538376734
C	3.5395005929530976	1.9153182267281432	0.0747407895885655
C	4.8994870473610934	3.1475374930851503	-2.0105420705454544
F	2.8520520018853475	3.7941623184869031	-2.9073160664606297
C	4.9151314385439138	1.8538553572017809	0.0428386245444072
F	2.9102038734355387	1.2805096392674411	1.0550283452233671
C	5.5854756689821405	2.4819697173207893	-1.0004945044868014
H	5.4128688247025076	3.6334553040890594	-2.8260777667273023
H	5.4447409994517812	1.3242130484131411	0.8202090115166690
C	7.0165147666586911	2.4333469184579455	-1.0407916458577340
N	-5.9694266993975580	2.5472727121043754	0.1242258587381089
N	8.1634210996276391	2.3924929653389260	-1.0742106706604417
